# The E3 ubiquitin ligase Cul5 regulates hematopoietic stem cell function for steady-state hematopoiesis in mice

**DOI:** 10.1172/JCI180913

**Published:** 2025-06-26

**Authors:** Siera A. Tomishima, Dale D. Kim, Nadia Porter, Ipsita Guha, Asif A. Dar, Yohaniz Ortega-Burgos, Jennifer Roof, Hossein Fazelinia, Lynn A. Spruce, Christopher S. Thom, Robert L. Bowman, Paula M. Oliver

**Affiliations:** 1 Department of Pathology and Laboratory Medicine, Perelman School of Medicine, University of Pennsylvania, Philadelphia, Pennsylvania, USA.; 2 Division of Protective Immunity,; 3 Division of Cell Pathology, and; 4 Division of Neonatology, Children’s Hospital of Philadelphia, Philadelphia, Pennsylvania, USA.; 5 Department of Pediatrics and; 6 Department of Cancer Biology, Perelman School of Medicine, University of Pennsylvania, Philadelphia, Pennsylvania, USA.

**Keywords:** Hematology, Immunology, Stem cells, Bone marrow differentiation, Hematopoietic stem cells, Ubiquitin-proteosome system

## Abstract

The balance of hematopoietic stem cell (HSC) self-renewal versus differentiation is essential to ensure long-term repopulation capacity while allowing response to events that require increased hematopoietic output. Proliferation and differentiation of HSCs and their progeny are controlled by the JAK/STAT pathway downstream of cytokine signaling. E3 ubiquitin ligases, like Cullin 5 (CUL5), can regulate JAK/STAT signaling by degrading signaling intermediates. Here we report that mice lacking CUL5 in hematopoietic cells (Cul5^Vav-Cre^) have increased numbers of hematopoietic stem and progenitor cells (HSPCs), splenomegaly, and extramedullary hematopoiesis. Differentiation in Cul5^Vav-Cre^ mice is myeloid- and megakaryocyte-biased, resulting in leukocytosis, anemia, and thrombocytosis. Cul5^Vav-Cre^ mice had increased HSC proliferation and circulation, associated with a decrease in CXCR4 surface expression. In bone marrow cells, we identified LRRC41 coimmunoprecipitated with CUL5, and vice versa, supporting that CRL5 forms a complex with LRRC41. We identified an accumulation of LRRC41 and STAT5 in Cul5^Vav-Cre^ HSCs during IL-3 stimulation, supporting their regulation by CUL5. Whole-cell proteome analysis of HSPCs from Cul5^Vav-Cre^ bone marrow identified upregulation of many STAT5 target genes and associated pathways. Finally, JAK1/2 inhibition with ruxolitinib normalized hematopoiesis in Cul5^Vav-Cre^ mice. These studies demonstrate the function of CUL5 in HSC function, stem cell fate decisions, and regulation of IL-3 signaling.

## Abstract 2

The E3 ubiquitin ligase Cul5 limits pSTAT5 signaling downstream of IL-3 to regulate HSC proliferation, cell fate, and steady-state hematopoiesis.

## Graphical abstract

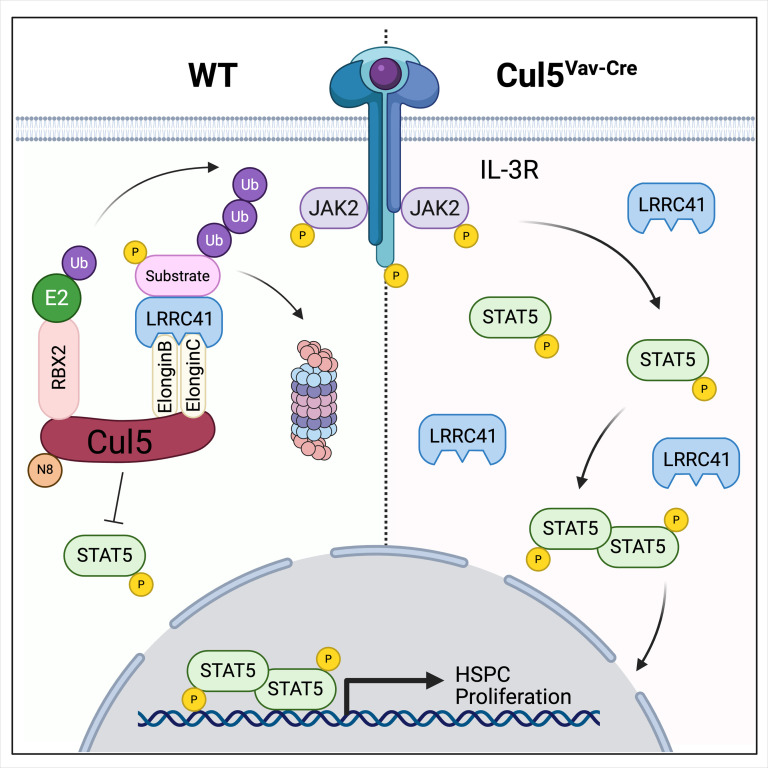

## Introduction

During homeostatic differentiation, hematopoietic stem cells (HSCs) are largely quiescent, maintaining a lifelong stem cell reserve in the bone marrow (1). Stressors such as infection can drive HSCs to switch to “emergency hematopoiesis,” favoring myeloid-biased differentiation over self-renewal to fulfill increased immune cell demand. Once this stress is resolved, HSCs return to their normal proliferative balance (2). HSCs rely on several cytokines that influence their activity. Cytokine signaling by thrombopoietin (TPO), stem cell factor (SCF), and interleukin-3 (IL-3) has diverse and synergistic roles in HSC function, including proliferation, differentiation, and survival (3–7). In addition to effects on steady-state hematopoiesis, IL-3 can mediate myeloid-biased differentiation during emergency events (8). There is also evidence that IL-3 may have negative effects on long-term survival of HSCs (9, 10). Conditions such as chronic inflammation or malignant hematopoiesis disrupt the typical balance of blood cell production, which can lead to stem cell exhaustion and bone marrow failure (11). For these reasons, proliferation and cell fate decisions of HSCs are tightly regulated by multiple mechanisms, including cytokine signaling.

Cytokine receptor binding initiates a signaling cascade, resulting in the activation of the JAK/STAT signaling pathway, and the transcription of STAT target genes (12). JAK/STAT signaling in hematopoietic cells is negatively regulated by suppressor of cytokine signaling (SOCS) proteins (13, 14). SOCS1, SOCS2, SOCS3, and CIS have been shown to inhibit JAK/STAT signaling downstream of multiple cytokines required for hematopoiesis, including EPO, TPO, IL-3, IL-6, G-CSF, SCF, and GM-CSF (15–20). After cytokine stimulation, phosphorylated STATs induce transcription of SOCS proteins as a negative-feedback mechanism. SOCS proteins can directly inhibit JAKs through their kinase inhibitory region (SOCS1 and SOCS3) (21, 22) or can assemble with E3 ubiquitin ligases (E3s) to target JAKs for ubiquitination and proteasomal degradation (23). SOCS proteins can act as substrate receptors for Cullin 5 (CUL5), a scaffold protein that facilitates the assembly of Cullin–RING ligase complex 5 (CRL5). In this capacity, SOCS box–containing substrate receptors confer specificity to CRL5 by recruiting target proteins for ubiquitination and proteasomal degradation (24–26).

The specificity of CRL5 complexes and their substrates is cell type and context dependent. This necessitates study of the function of CRL5 complexes in vivo to more fully understand how they are regulated and perturbed by disease. Despite the well-described roles of SOCS proteins in hematopoietic stem and progenitor cells (HSPCs), the extent to which their function relies on interaction with CUL5 remains poorly understood. CUL5 downregulation has been implicated as a marker of poor prognosis in patients with uterine cancer (27), lung cancer (28, 29), kidney cancer (30), and a subset of B cell chronic lymphocytic leukemia (31). A recent study found that CUL5 limits the production of megakaryocyte-biased HSCs in an IL-3–dependent manner (32). However, the potential substrate receptors and substrates in the CRL5 complex responsible for this regulation were not explored.

In this study, we assessed the function of CUL5 during hematopoiesis by conditional deletion in hematopoietic cells. We found that Cul5^Vav-Cre^ mice had excessive HSPC proliferation in both bone marrow and spleen, myeloid- and megakaryocyte-biased hematopoiesis, and sustained phosphorylated STAT5 (p-STAT5) signaling downstream of IL-3 stimulation. JAK1/2 inhibition reversed splenomegaly and improved myeloid-lymphoid balance, supporting that abnormal hematopoiesis was caused by hyperactive JAK/STAT signaling. These results reveal a role for CUL5 in the regulation of HSC proliferation and differentiation, and in regulation of p-STAT5 and IL-3 receptor signaling. These insights demonstrate potential therapeutic targets for the treatment of hematopoietic malignancies and other immune-related illnesses.

## Results

### CUL5 deficiency drives dysregulated hematopoiesis.

**Figure 1:**
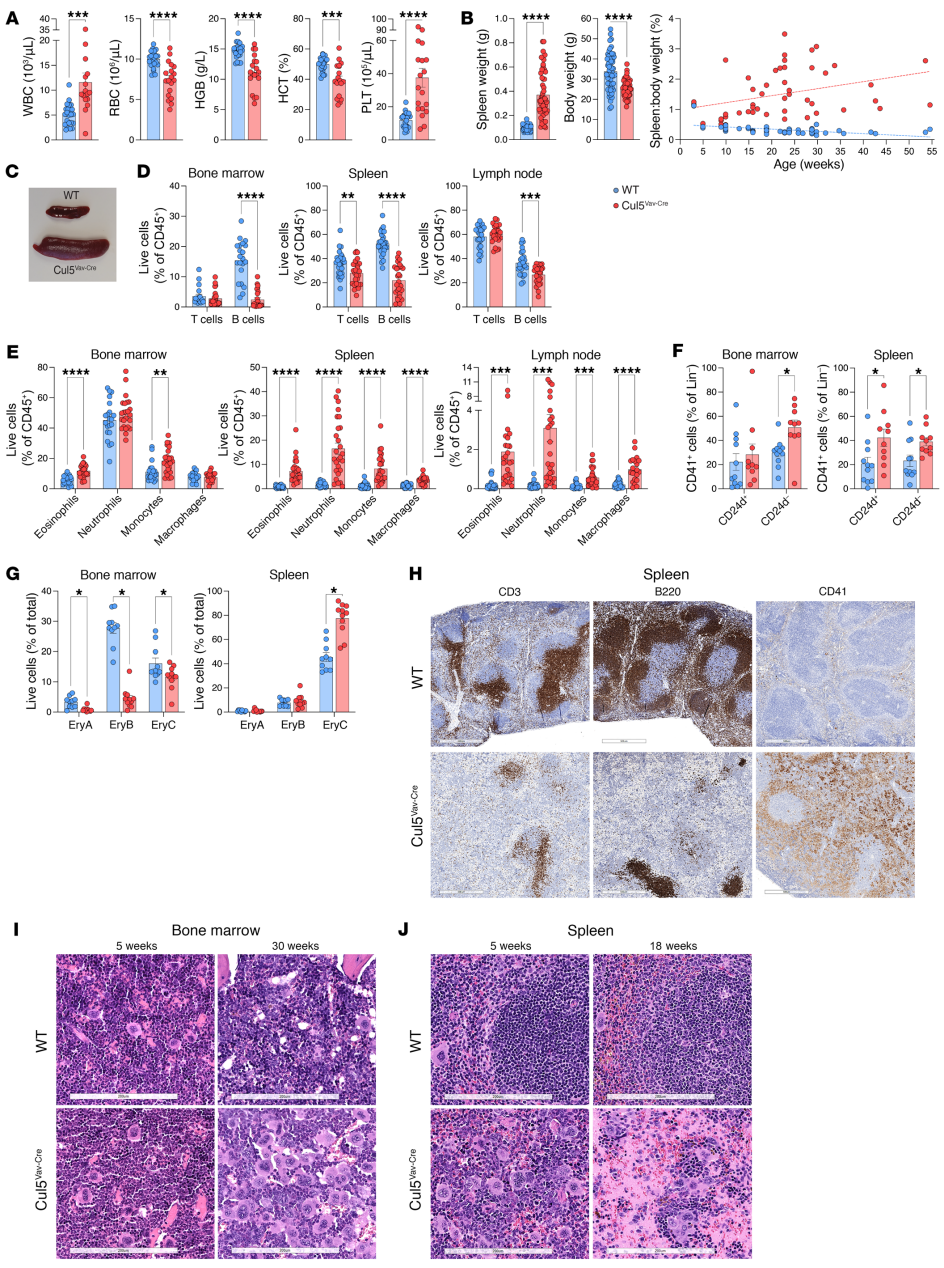
Cul5^Vav-Cre^ mice have lineage-biased hematopoiesis. (**A**) Complete blood count analysis of WT and Cul5^Vav-Cre^ mice (*n* ≥ 23). (**B**) Spleen weight, body weight, and spleen/body weight ratio of WT and Cul5 mice (*n* ≥ 48). (**C**) Representative image of WT and Cul5^Vav-Cre^ spleens. (**D**–**G**) Percentages of lymphoid cells (**D**), myeloid cells (**E**), megakaryocytes and platelets (**F**), and erythroid progenitors (**G**) in WT and Cul5^Vav-Cre^ bone marrow, spleen, and/or lymph nodes (*n* ≥ 10). (**H**) Representative IHC of CD3 (T cells), B220 (B cells), and CD41 (platelets and megakaryocytes) in WT and Cul5^Vav-Cre^ spleens (*n* = 3). Scale bars: 500 μm. (**I** and **J**) Representative H&E of WT and Cul5^Vav-Cre^ bone marrow (femur) at 5 and 30 weeks (**I**) and spleen at 5 and 18 weeks (**J**) (*n* ≥ 7). Scale bars: 200 μm. The following tests were used to determine significance: (**A** and **B**) unpaired 2-tailed *t* test; (**D**–**G**) unpaired *t* test with Holm-Šidák correction. *P < 0.05; **P < 0.01; ***P < 0.001; ****P < 0.0001. Male and female mice aged 5–55 weeks were analyzed for **A**–**G**.

CUL5 is known to limit cytokine signaling in multiple cell types, including CD4^+^ T cells (33), but its role in other hematopoietic cells is poorly understood. To study the function of CUL5 in immune cells, we generated Cul5^fl/fl^ Vav-Cre (Cul5^Vav-Cre^) mice, which results in deletion of CUL5 in all hematopoietic cells. Mice were born at expected Mendelian frequencies and were indistinguishable from their WT littermates through weaning. However, as early as 5 weeks of age, Cul5^Vav-Cre^ mice developed leukocytosis, thrombocythemia, anemia, and low hemoglobin and hematocrit (Figure 1A). They developed splenomegaly and had reduced body weight compared with their WT littermates, which resulted in increased spleen to body weight ratio (Figure 1, B and C). Despite increased spleen size, overall numbers of live cells in the spleen counted after red blood cell lysis were not different between WT and Cul5^Vav-Cre^ mice. However, cell numbers in the spleen before red blood cell lysis were significantly higher in Cul5^Vav-Cre^ mice, supporting that elevated red blood cell numbers might partly explain the increased spleen size (Supplemental Figure 1A; supplemental material available online with this article; https://doi.org/10.1172/JCI180913DS1). In contrast, cell numbers in the bone marrow were increased in Cul5^Vav-Cre^ mice after, but not before, red blood cell lysis (Supplemental Figure 1B).

To assess the differences in numbers of immune cells in more detail, we analyzed cells isolated from primary and secondary lymphoid organs using flow cytometry (Supplemental Figure 1C). Cul5^Vav-Cre^ mice showed a reduction in B cell numbers and frequencies in spleen, bone marrow, and lymph nodes as well as a reduction in T cells in the spleen (Figure 1D and Supplemental Figure 1, D and E). In contrast, Cul5^Vav-Cre^ mice had increased frequencies and numbers of monocytes, macrophages, eosinophils, and neutrophils in spleen and lymph nodes compared with littermate controls (Figure 1E and Supplemental Figure 1F). Megakaryocyte and platelet frequencies (Lin^–^ CD41^+^ CD42d^+/–^) were increased in Cul5^Vav-Cre^ spleen and bone marrow, while mature erythrocyte frequencies (Ery C: CD71^+^ Ter119^+^ FSC^lo^) were increased in the spleen. All Ter119^+^ erythroid populations (Ery A: CD71^+^ FSC^hi^; Ery B: CD71^+^ FSC^lo^) were decreased in bone marrow of Cul5^Vav-Cre^ mice (Figure 1, F and G).

Given the observed alterations in immune cell numbers, we assessed tissue architecture using histology. Immunohistochemical analysis of spleen also showed fewer B cells (B220^+^) and T cells (CD3^+^) as well as increased platelets and megakaryocytes (CD41^+^) (Figure 1H). Histological analysis of spleen revealed disrupted splenic architecture with large acellular areas and megakaryocytic hyperplasia that was also evident in the bone marrow (Figure 1, I and J).

### CUL5 deficiency results in an increase in HSCs and myeloid/erythroid progenitors.

**Figure 2:**
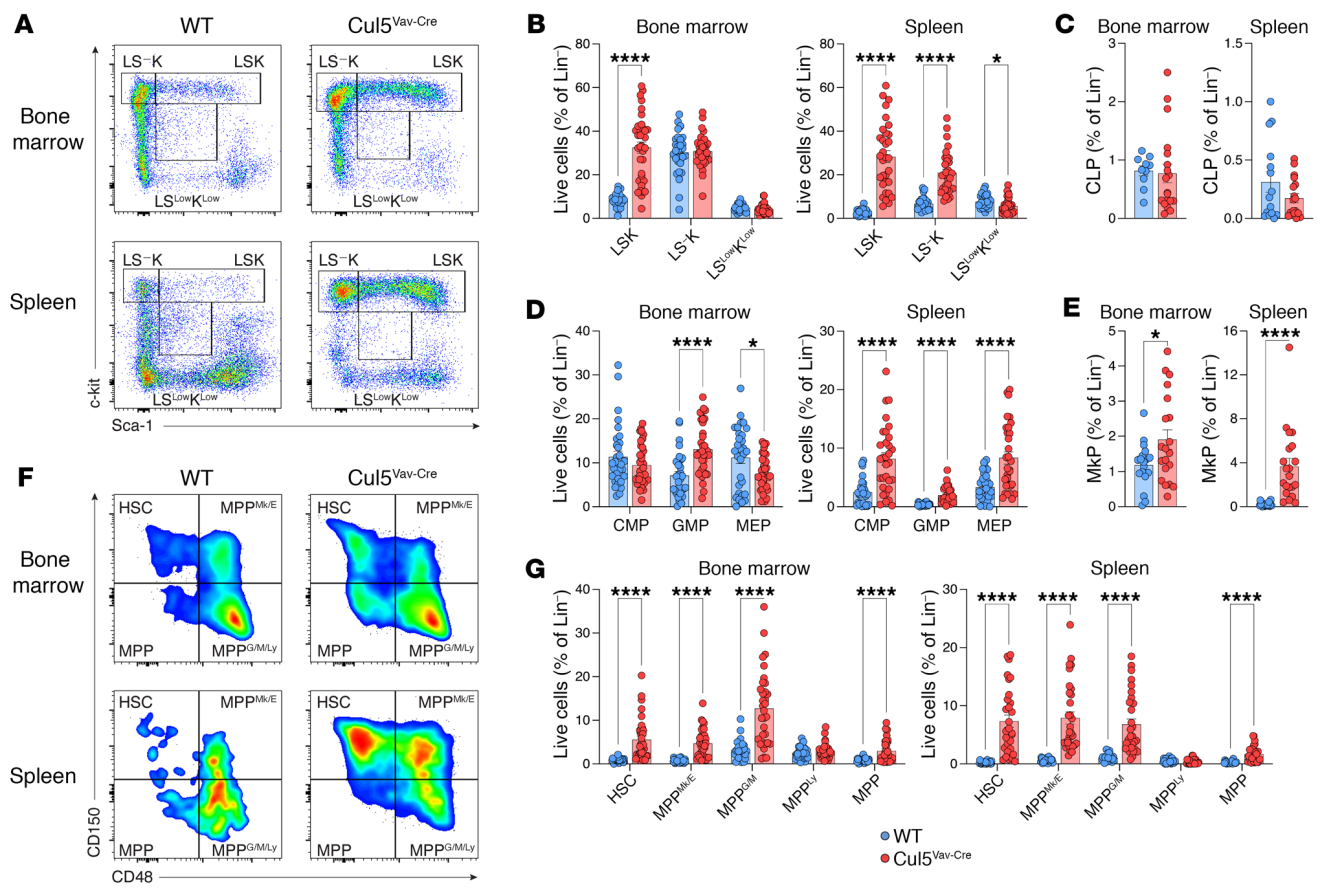
Cul5 regulates HSPC proportions. (**A**) Representative flow plots of lineage-negative populations in WT and Cul5^Vav-Cre^ spleen and bone marrow. (**B**–**E** and **G**) Percentage of lineage-negative populations (**B**), common lymphoid progenitors (CLPs) (**C**), LS^–^K populations (**D**), MkPs (**E**), and LSK populations (**G**) of WT and Cul5^Vav-Cre^ spleen and bone marrow (*n* ≥ 20). (**F**) Representative flow plots of LSK populations in WT and Cul5^Vav-Cre^ spleen and bone marrow. The following tests were used to determine significance: (**B**, **D**, and **G**) unpaired *t* test with Holm-Šidák correction; (**C** and **E**) unpaired *t* test. *P < 0.05; ****P < 0.0001. Male and female mice aged 5–55 weeks were analyzed for **A**–**G**.

Given the alteration in the proportion of multiple populations of immune cells in primary and secondary lymphoid organs, we looked at HSPC populations in the spleen and bone marrow to determine whether these cell types were impacted by CUL5 loss. We used flow cytometry to assess lineage-negative (Lin^–^), Lin^–^ Sca-1^+^ c-kit^+^ (LSK), and Lin^–^ Sca-1^–^ c-kit^+^ (LS^–^K) populations (Supplemental Figure 2A). Cul5^Vav-Cre^ mice had drastically increased LSK cells in the spleen and bone marrow, as well as increased LS^–^K cells in the spleen (Figure 2, A and B, and Supplemental Figure 2B). We further subdivided these populations to see whether there was a bias to a particular lineage during hematopoiesis. Within the spleen, there was an increase in all LS^–^K populations, including common myeloid progenitors (CMP: LS^−^K CD16/32^−^ CD34^+^), granulocyte macrophage progenitors (GMP: LS^−^K CD16/32^+^ CD34^+^), and megakaryocyte erythroid progenitors (MEP: LS^−^K CD16/32^−^ CD34^−^) (Figure 2D and Supplemental Figure 2D). Megakaryocyte progenitors (MkP: LS^–^K CD34^–^ CD16/32^–^ CD150^+^ CD41^+^) were increased in both the bone marrow and spleen (Figure 2E and Supplemental Figure 2E). To look at uncommitted cells, we further delineated LSKs into HSCs and multipotent progenitor (MPP) populations as defined by Challen et al. (34) using the gating scheme from Eich et al. (35): HSCs (LSK CD150^+^ CD48^−^), MPPs (LSK CD150^−^ CD48^−^), MPP^Mk/E^ (LSK CD150^+^ CD48^+^), MPP^G/M^ (LSK CD150^−^ CD48^+^ CD135^−^), and MPP^Ly^ (LSK CD150^−^ CD48^+^ CD135^+^). HSCs and MPPs as well as erythroid-megakaryocyte-biased MPP^Mk/E^ and myeloid-biased MPP^G/M^ cells were increased in both spleen and bone marrow (Figure 2, F and G, and Supplemental Figure 2F). Common lymphoid progenitors and MPP^Ly^ populations, which give rise to lymphocytes (36), were not different in spleen and bone marrow of Cul5^Vav-Cre^ mice (Figure 2, C and G, and Supplemental Figure 2, C and F).

### CUL5 inhibits proliferation of HSPCs.

**Figure 3:**
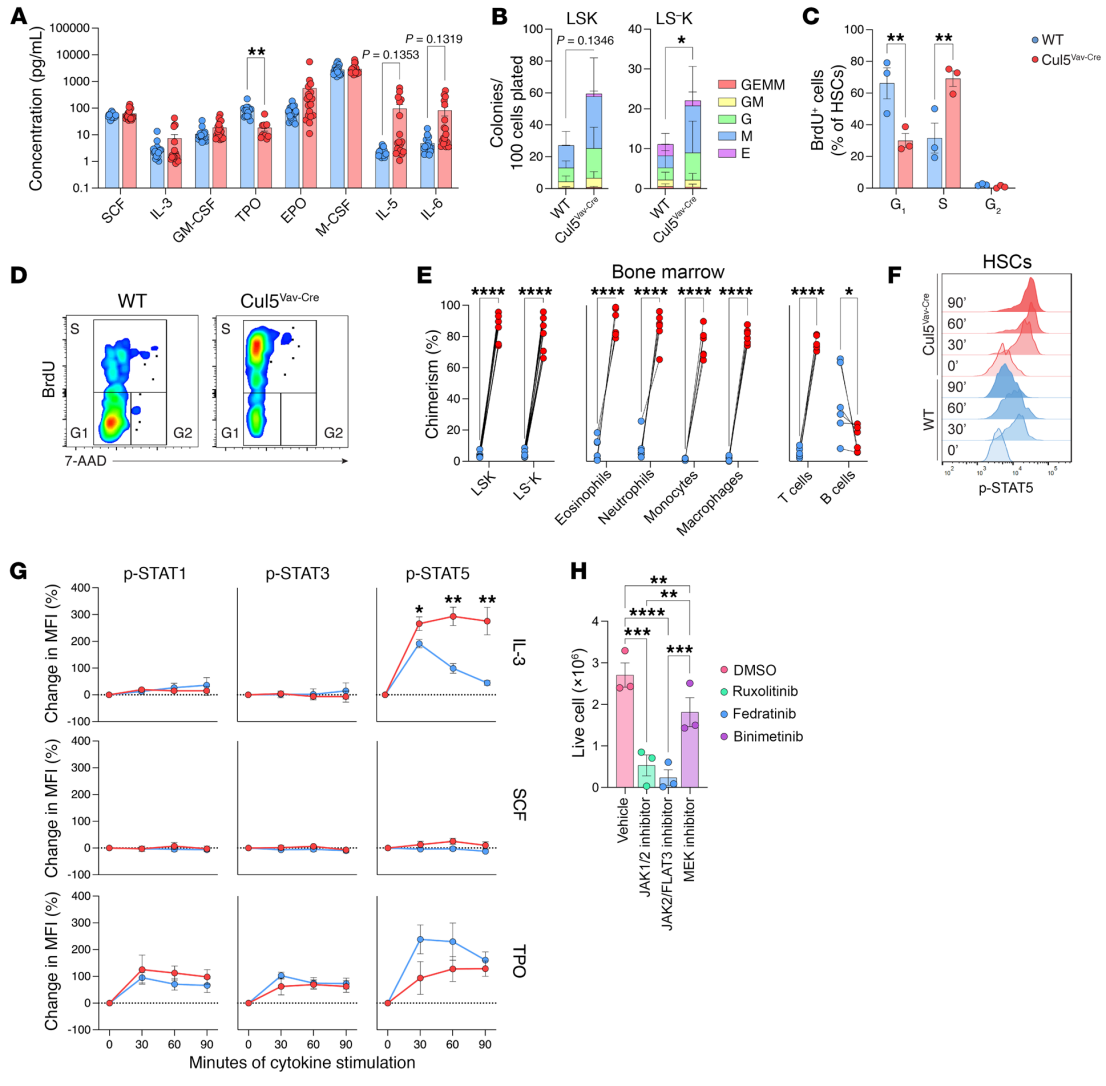
Cul5-deficient HSPCs exhibit cytokine hypersensitivity. (**A**) Serum concentrations of cytokines from WT and Cul5^Vav-Cre^ mice (*n* = 18). (**B**) CFU counts from LSK and LS^–^K sorted from WT and Cul5^Vav-Cre^ spleen (*n* = 3 biological replicates; 3 technical replicates per experiment). (**C**) Percentage of WT and Cul5^Vav-Cre^ bone marrow CD34^lo^ HSCs in S, G_1_, and G_2_ phase (*n* = 3; >280 HSCs analyzed per sample). (**D**) Representative flow plots of BrdU and 7-aminoactinomycin D (7-AAD) in WT and Cul5^Vav-Cre^ bone marrow HSCs. (**E**) Percentage chimerism of HSPCs and myeloid and lymphoid cells in bone marrow of WT and Cul5^Vav-Cre^ competitive bone marrow transplants at 5–9 weeks after reconstitution (*n* ≥ 6). (**F**) Representative histogram of p-STAT5 induction in WT and Cul5^Vav-Cre^ bone marrow HSCs after IL-3 stimulation (20 ng/mL). (**G**) Percentage change of p-STAT1, p-STAT3, or p-STAT5 MFI in WT and Cul5^Vav-Cre^ HSCs after IL-3, TPO, or SCF stimulation (20 ng/mL) (*n* ≥ 3). (**H**) Live cell numbers from CFUs of Cul5^Vav-Cre^ spleen cells in the presence of DMSO, ruxolitinib, fedratinib, or binimetinib (1 μM; *n* = 3). The following tests were used to determine significance: (**A**, **C**, **E**, and **G**) unpaired *t* test with Holm-Šidák correction; (**B**) paired *t* test; (**H**) 2-way ANOVA with Holm-Šidák correction. *P < 0.05; **P < 0.01; ***P < 0.001; ****P < 0.0001. Male and female mice of the following ages were analyzed: (**A**) 5–55 weeks; (**B**) 9–24 weeks; (**C** and **D**) 21–29 weeks; (**F** and **G**) 22–55 weeks; (**H**) 23–30 weeks.

Given the increased numbers of multiple hematopoietic progenitor populations, we sought to test whether Cul5^Vav-Cre^ mice had altered levels of cytokines that could drive an increased expansion of HSPCs. To test this, we assessed serum cytokine levels in WT and Cul5^Vav-Cre^ mice by flow cytometry using a bead-based immunoassay. While we identified trends toward increased IL-5 and IL-6 in some Cul5^Vav-Cre^ mice, most showed no significant differences across a range of cytokines required for HSPC and myeloid cell survival and proliferation (Figure 3A and Supplemental Figure 3A). We additionally identified increased EPO levels and a trend toward decreased levels of TPO in Cul5^Vav-Cre^ mice, which is consistent with decreased circulating red blood cells (37) and increase in platelet counts in blood and spleen (38).

We next tested whether the increase in HSPC numbers in Cul5^Vav-Cre^ mice was due to altered responses to cytokines. We compared the ability of WT and CUL5-deficient cells to form colonies in vitro in methylcellulose medium with IL-3, SCF, IL-6, and EPO. To normalize for the increased proportion of progenitors in Cul5^Vav-Cre^ spleen, we sorted LSK and LS^–^K cells from spleens of WT and Cul5^Vav-Cre^ mice and plated an equal number of cells in colony-forming unit (CFU) assays. We found that more colonies formed from the Cul5^Vav-Cre^ LSK and LS^–^K spleen cells compared with WT counterparts (Figure 3B), suggesting that CUL5 regulates sensitivity to cytokines and thus impacts their proliferation and/or survival.

To determine whether the elevated number of HSPCs in Cul5^Vav-Cre^ mice was due to an increase in proliferation, we exposed mice to BrdU in their drinking water and then assessed for BrdU incorporation. Cul5^Vav-Cre^ bone marrow CD34^lo^ HSCs had increased numbers of cells in S phase (BrdU^+^) compared with WT cells (Figure 3, C and D), demonstrating that loss of CUL5 increases proliferation of HSCs.

To test whether CUL5-deficient HSPCs could outcompete WT cells in seeding and repopulating in vivo, we performed competitive bone marrow transplants. We transplanted equal mixtures of WT (CD45.1) and Cul5^Vav-Cre^ (CD45.2) bone marrow cells into lethally irradiated hosts (CD45.1×CD45.2). After reconstitution, mice were assessed for their frequencies of progenitors and committed progeny. Cul5^Vav-Cre^-derived progenitors (LSK and LS^−^K) significantly outnumbered WT cells in both bone marrow and spleen (Figure 3E and Supplemental Figure 3B). Accordingly, Cul5^Vav-Cre^ myeloid populations vastly outnumbered those from WT in spleen, bone marrow, and lymph node (Figure 3E and Supplemental Figure 3, B and C). We observed significantly less Cul5^Vav-Cre^ chimerism in B cells in bone marrow, spleen, and lymph node, consistent with B cell deficiencies in Cul5^Vav-Cre^ mice. T cells were more variable, and differences were observed depending on the tissue (Figure 3E and Supplemental Figure 3, B and C). These data support an HSPC-intrinsic role for CUL5 in short-term hematopoiesis.

### CUL5 limits p-STAT5 signaling following stimulation in vitro.

Owing to the known role of CUL5 in targeting JAKs for ubiquitination, we next wanted to determine whether Cul5^Vav-Cre^ HSPCs demonstrated increased JAK/STAT signaling. We looked at the induction of p-STAT5 downstream of stimulation with cytokines known to regulate HSC proliferation and survival — IL-3, TPO, and SCF. While there was no statistical difference in phosphorylation of STAT1, STAT3, or STAT5 between WT and Cul5^Vav-Cre^ HSPCs following TPO and SCF, we found that multiple subsets of Cul5^Vav-Cre^ HSPCs showed increased p-STAT5 following IL-3 stimulation. The most striking differences were seen in HSCs (Figure 3, F and G) and MEPs (Supplemental Figure 3D), but modest differences were seen in CMPs/GMPs, MPP^Mk/E^, MPP^G/M^, and MPPs (Supplemental Figure 3D). Given the sustained signal of p-STAT5 in CUL5-deficient HSPCs, we tested whether this caused increased proliferation of these cells. We performed a CFU assay with spleen cells from Cul5^Vav-Cre^ mice with the addition of ruxolitinib, a JAK1/2 inhibitor, fedratinib, a JAK2/FLT3 inhibitor, and binimetinib, a MEK inhibitor. We used binimetinib to test whether MEK/ERK signaling, which is downstream of IL-3 receptor stimulation but results in the transcription of target genes different from those transcribed by STAT5 (39, 40), was also contributing to the differences in Cul5^Vav-Cre^ HSPCs. Ruxolitinib and fedratinib both significantly inhibited cell growth compared with vehicle, while binimetinib only slightly reduced cell growth (Figure 3H). This indicates that CUL5 regulation of JAK/STAT signaling regulates proliferation and expansion of HSPCs. Given the pronounced differences in STAT5 phosphorylation in vitro as well as the increased proliferation of HSCs observed in vivo, we opted to focus further analyses on HSCs and LSKs.

### CUL5-deficient LSKs have impaired homing and repopulation capacity.

We next wanted to see whether Cul5^Vav-Cre^ cells had the ability to maintain long-term repopulation capacity in serially transplanted mice. We transplanted whole bone marrow mixed at a 10:1 ratio of WT to Cul5^Vav-Cre^ cells into lethally irradiated recipients, which resulted in an average of 65% WT and 35% Cul5^Vav-Cre^ LSK chimerism. Primary recipients had increased LSK contribution from Cul5^Vav-Cre^ donors with 6 of 8 mice having over 80% Cul5^Vav-Cre^ contribution. Secondary recipients did not have significantly different chimerism with 7 of 14 mice having over 50% Cul5^Vav-Cre^ contribution (Supplemental Figure 4A). Chimerism of mature myeloid cells from the bone marrow was equal in primary recipients, but favored WT contribution by the secondary transplant. B and T cell chimerism from the bone marrow was heavily skewed toward WT in both primary and secondary recipients, consistent with lymphoid deficiencies in Cul5^Vav-Cre^ mice (Supplemental Figure 4B). Despite mixed chimerism in primary and secondary recipients, we found that increased HSC proportion was correlated with increased Cul5^Vav-Cre^ contribution (Supplemental Figure 4, C and D). This suggested that despite having low contribution to mature cells in the bone marrow, Cul5^Vav-Cre^ HSCs maintain increased proliferation rates even after secondary transplantation.

Since Cul5^Vav-Cre^ mice have higher proportions of HSPCs than WT mice, we performed competitive transplants using equal numbers of sorted HSCs (LSK CD150^+^ CD48^–^) or MPPs (LSK CD150^–^ CD48^+/–^). We analyzed peripheral blood every 4 weeks to assess the chimerism of mature immune cells. At 4 weeks, myeloid cells (CD11b^+^) were almost exclusively derived from Cul5^Vav-Cre^ progenitors in the MPP recipient mice. However, overall chimerism in the HSC recipients slightly skewed toward WT through the duration of the experiment (Supplemental Figure 4E). But as expected, neither WT or Cul5^Vav-Cre^ MPPs showed prolonged repopulation capacity (Supplemental Figure 4F).

**Figure 4:**
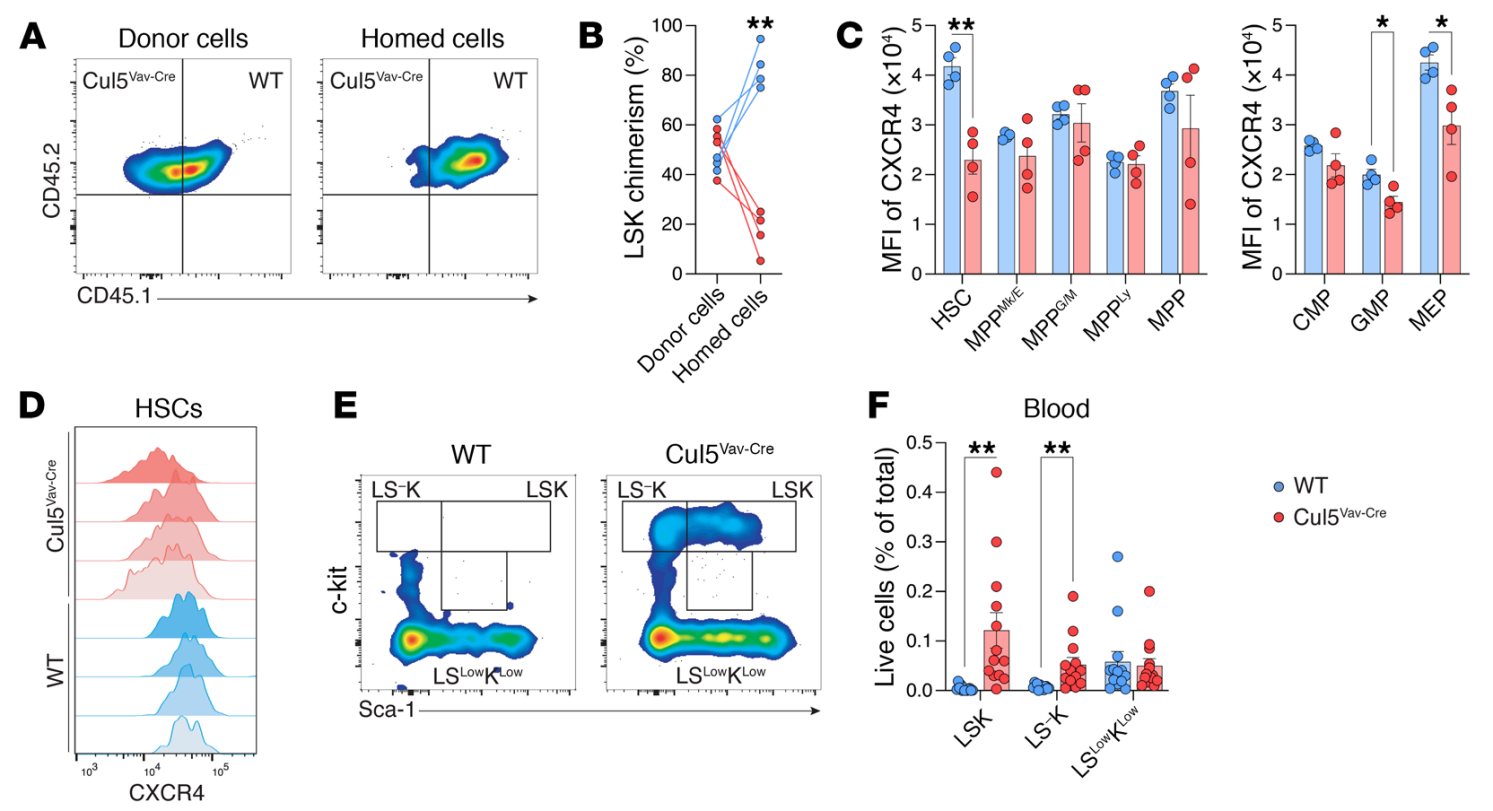
Cul5-deficient LSKs have bone marrow homing impairment. (**A**) Representative flow plots of CD45.1 and CD45.2 expression on LSKs from donor cells and cells homed to the bone marrow in recipient mice. (**B**) Percentage chimerism of LSKs in the bone marrow of recipient mice after 15 hours (*n* = 4). (**C**) MFI of CXCR4 in WT and Cul5^Vav-Cre^ lineage-negative populations in bone marrow (*n* = 4). (**D**) Histograms of CXCR4 in WT and Cul5^Vav-Cre^ bone marrow HSCs (*n* = 4). (**E**) Representative flow plots of lineage-negative cells in blood from WT and Cul5^Vav-Cre^ mice. (**F**) Percentage of lineage-negative cells in blood from WT and Cul5^Vav-Cre^ mice (*n* = 13). The following test was used to determine significance: (**B**, **C**, and **E**) unpaired *t* test with Holm-Šidák correction. *P < 0.05; **P < 0.01. Male and female mice of the following ages were analyzed: (**A**–**D**) 16–39 weeks; (**E** and **F**) 7–34 weeks.

To investigate why transplanting limiting numbers of Cul5-deficient HSCs did not recapitulate whole bone marrow transplants with saturating cell numbers, we assessed the homing capacity of lineage-negative cells from WT and Cul5^Vav-Cre^ mice. We isolated lineage-negative cells from WT and Cul5^Vav-Cre^ bone marrow, mixed them to achieve a roughly 1:1 ratio of HSCs, and transplanted them into lethally irradiated recipients. After 15 hours, we collected bone marrow from recipients and assessed for presence of transplanted stem cells. We found fewer Cul5^Vav-Cre^ LSKs than WT, indicating that they have reduced homing capacity to the bone marrow (Figure 4, A and B). We hypothesized that the homing defect might be a result of altered cytokine sensing and assessed expression of CXCR4 on bone marrow cells, a chemokine receptor that is essential in stem cell homing (41). HSCs from Cul5^Vav-Cre^ bone marrow had significantly reduced CXCR4 expression on their surface compared with WT HSCs (Figure 4, C and D). One consequence of CXCR4 loss or inhibition is increased mobilization of HSPCs (42). Consistent with this, we observed that both LSK and LS^–^K populations were increased in blood of Cul5^Vav-Cre^ mice compared with WT (Figure 4, E and F). Collectively these data demonstrate that Cul5^Vav-Cre^ HSCs downregulate CXCR4, resulting in increased peripheral blood mobilization, and consequently decreased fitness in comparison with WT cells in competitive transplant assays due to defects in bone marrow homing and retention.

### CUL5 binds STAT5 downstream of IL-3, SCF, and TPO stimulation.

**Figure 5:**
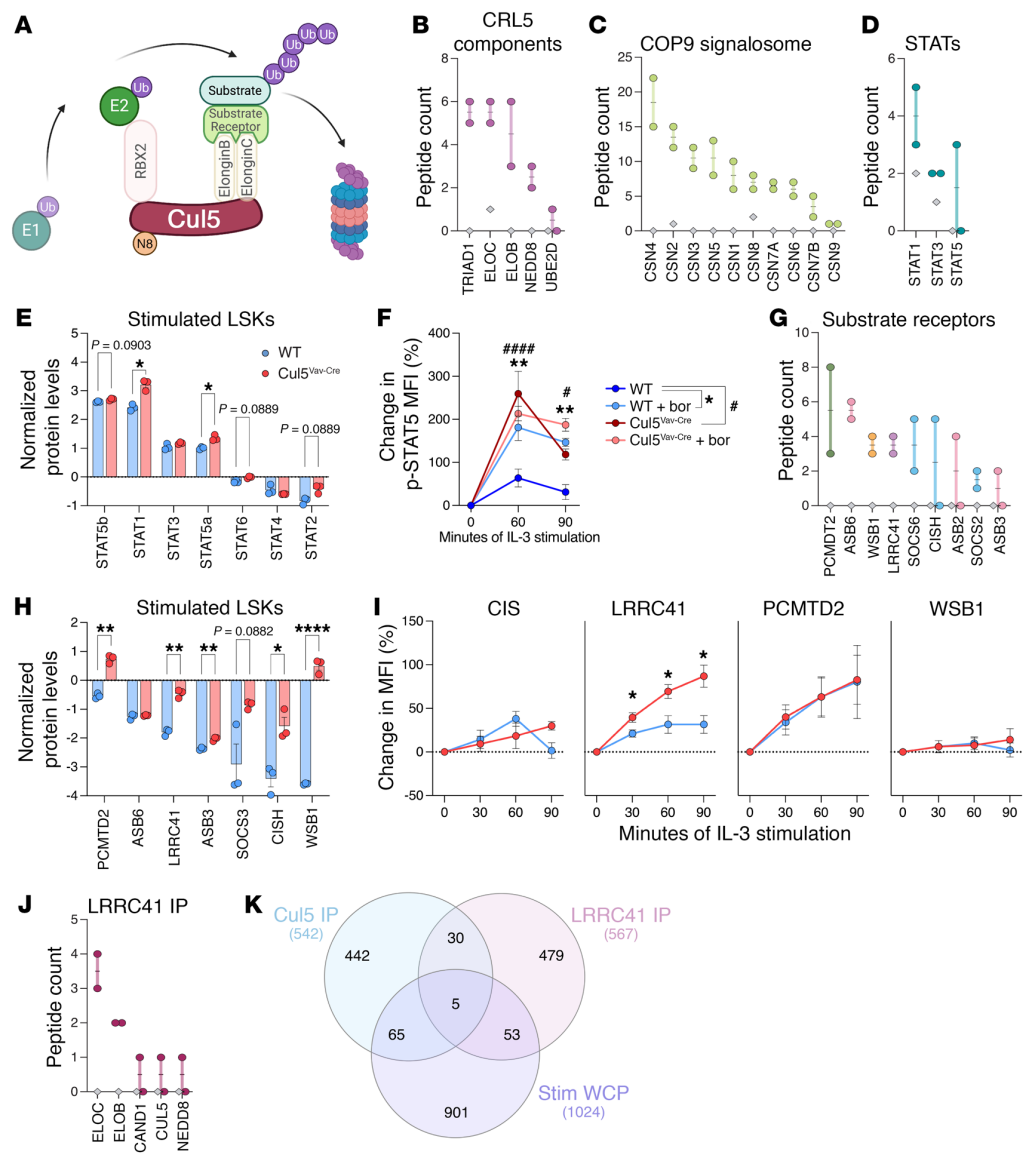
Cul5 binds STAT5 and LRRC41 in hematopoietic cells. (**A**) Depiction of CRL5 complex in substrate ubiquitination. (**B**–**D**) Peptide counts of CRL5 components (**B**), COP9 signalosome proteins (**C**), and STATs (**D**) coimmunoprecipitated with CUL5 (*n* = 2) or IgG (*n* = 1) from cultured WT HSPCs. (**E**) Normalized protein quantification of STATs in stimulated WT and Cul5^Vav-Cre^ LSKs (*n* = 3). (**F**) Percentage change of p-STAT5 MFI in untreated or bortezomib-treated (500 nM) WT and Cul5^Vav-Cre^ HSCs stimulated with IL-3 (50 ng/mL; *n* = 3). (**G**) Peptide counts of substrate receptors coimmunoprecipitated with CUL5 (*n* = 2) or IgG (*n* = 1) from cultured WT HSPCs. (**H**) Normalized protein quantification of substrate receptors in stimulated WT and Cul5^Vav-Cre^ LSKs (*n* = 3). (**I**) Percentage change of CIS, PCMTD2, LRRC41, and WSB1 MFI in IL-3–stimulated (20 ng/mL) WT and Cul5^Vav-Cre^ HSCs (*n* ≥ 3). (**J**) Peptide counts of CRL5 components coimmunoprecipitated with LRRC41 (*n* = 2) or IgG (*n* = 2) from IL-3–stimulated bone marrow cells. (**K**) Venn diagram of proteins coimmunoprecipitated with CUL5, proteins coimmunoprecipitated with LRRC41, and proteins increased in stimulated Cul5^Vav-Cre^ LSKs over WT LSKs. In **B**–**D** and **G**, circles represent Cul5 immunoprecipitation (IP), and diamonds represent IgG IP. In **J**, circles represent LRRC41 IP, and diamonds represent IgG IP. The following tests were used to determine significance: (**E**, **H**, and **I**) unpaired *t* test with Holm-Šidák correction; (**F**) 2-way ANOVA with Holm-Šidák correction. *P < 0.05; **P < 0.01; ****P < 0.0001. ^#^*P* < 0.05; ^####^*P* < 0.0001. Male and female mice of the following ages were analyzed: (**B**–**D** and **G**) 8–14 weeks; (**E** and **H**) 23–38 weeks; (**F**) 11–13 weeks; (**H**) 8–14 weeks; (**I**) 22–55 weeks; (**J**) 11–19 weeks.

To determine the molecular mediators underlying dysregulated hematopoiesis in Cul5^Vav-Cre^ mice, we next sought to identify the other CRL5 complex components that might regulate JAK/STAT signaling in HSPCs. To identify CUL5 binding partners that limit STAT5 signaling, we immunoprecipitated CUL5 from HSPCs. Because of the limited number of LSKs per mouse (about 4 × 10^4^ to 1.5 × 10^5^) and the quantity of cells needed per replicate (5 × 10^7^), we were unable to directly query LSKs for immunoprecipitation. Instead, we expanded WT CD34^lo^ HSCs in culture with TPO and SCF as previously described (43), with the addition of IL-3. These cells expanded in culture are not exclusively HSCs, but include other HSPCs and immature myeloid populations. We then immunoprecipitated cell lysates with an anti-CUL5 antibody or an isotype control. Proteins bound to CUL5 or control antibody were identified by tandem mass spectrometry. To assess the quality and specificity of the CUL5 immunoprecipitation, we assessed binding of primary interactions with proteins known to complex with CUL5 (Figure 5A), including ARIH2, ELOB, ELOC, NEDD8 (Figure 5B), and COP9 signalosome components (Figure 5C). Interestingly, we found STAT1, STAT3, and STAT5 in complex with CUL5. While STAT1 and STAT3 were also bound to the isotype control, STAT5 was found uniquely in the CUL5 immunoprecipitation (Figure 5D). Whole-cell proteomes (WCPs) of WT and Cul5^Vav-Cre^ LSKs after stimulation with IL-3, SCF, and TPO for 1 hour revealed an increase in STAT1 and STAT5 in Cul5^Vav-Cre^ LSKs (Figure 5E), suggesting that CUL5 loss increased their stability.

Since it has been demonstrated that STAT5 is degraded by the proteasome following IL-3 stimulation (44), we wanted to test how p-STAT5 activity is influenced by CUL5 deficiency during proteasomal inhibition. We incubated HSCs with a proteasome inhibitor, bortezomib, for 30 minutes, followed by stimulation with IL-3 for 60–90 minutes. Bortezomib treatment of WT cells resulted in increased levels of p-STAT5, supporting that p-STAT5 is inhibited by proteasomal activity. In contrast, Cul5^Vav-Cre^ HSCs showed similar p-STAT5 levels after bortezomib treatment when compared with untreated counterparts (Figure 5F), implicating CUL5 as a causative component of p-STAT5 stability.

### CUL5 forms a complex with LRRC41 in bone marrow cells.

We then focused on interactions of potential substrate receptors. We identified 9 substrate receptors that coimmunoprecipitated with CUL5 (Figure 5G). CIS (15), SOCS2 (18), SOCS6 (45), PCMTD2 (46), and ASB2 (47–49) have known roles in regulating immune cell function and/or association with cancers, but the roles of ASB3, ASB6, WSB1, and LRRC41 in HSPCs are less understood. We sought to define the relationship between CUL5 and these potential substrate receptors. ASB2, SOCS2, and SOCS6 were not identified in either WCP analysis, supporting that these proteins are of very low abundance in LSKs or are more abundant in downstream progenitors or mature cell types present in the culture used for immunoprecipitation. We identified two ASB proteins, ASB3 and ASB6, and the levels of these proteins were similar in WT and Cul5^Vav-Cre^ LSKs. Intriguingly, we found that WSB1, PCMTD2, CIS, and LRRC41 protein abundance was over 2 times higher in Cul5^Vav-Cre^ compared with WT LSKs (Figure 5H). Substrate receptors for E3 ligases have been shown to accumulate in cells when the E3 ligases are absent (50). Thus, we reasoned that these 4 substrate receptors might work with CUL5 downstream of IL-3 signaling. We found that LRRC41, CIS, PCMTD2, and WSB1 were upregulated in HSCs following stimulation with IL-3, TPO, and SCF. While IL-3 stimulation resulted in similar WSB1, CIS, and PCMTD2 levels between WT and Cul5^Vav-Cre^ HSCs, LRRC41 levels were significantly increased in Cul5^Vav-Cre^ HSCs compared with WT HSCs (Figure 5I and Supplemental Figure 5A).

To assess whether LRRC41 is a substrate receptor for a CRL5 complex, we immunoprecipitated LRRC41 from WT bone marrow cells stimulated with IL-3. We found several CRL5 components bound to LRRC41, including CUL5, ELOB, ELOC, CAND1, and NEDD8 (Figure 5J and Supplemental Figure 5B). We further identified several proteasome subunits bound to LRRC41, supporting its involvement in proteasomal degradation (Supplemental Figure 5B). Next, we immunoprecipitated STAT5 from IL-3–stimulated bone marrow cells to assess overlap in proteins bound by CUL5, LRRC41, and STAT5 (Supplemental Figure 5C). We identified proteins that showed a 3-fold enrichment in the target immunoprecipitation compared with the IgG control as well as proteins that were significantly increased (*P* > 0.05) in stimulated Cul5^Vav-Cre^ LSKs over WT. One protein, LTA4H, was found in all 4 datasets, and 17 proteins were shared by at least 3 of the datasets (Figure 5K and Supplemental Figure 5, D and E). These data support that CUL5 utilizes LRRC41 as a substrate receptor following IL-3 stimulation and that CUL5, LRRC41, and STAT5 are regulated by or are regulating overlapping pathways.

### Cul5^Vav-Cre^ LSK proteomes are enriched for STAT5 target genes.

**Figure 6:**
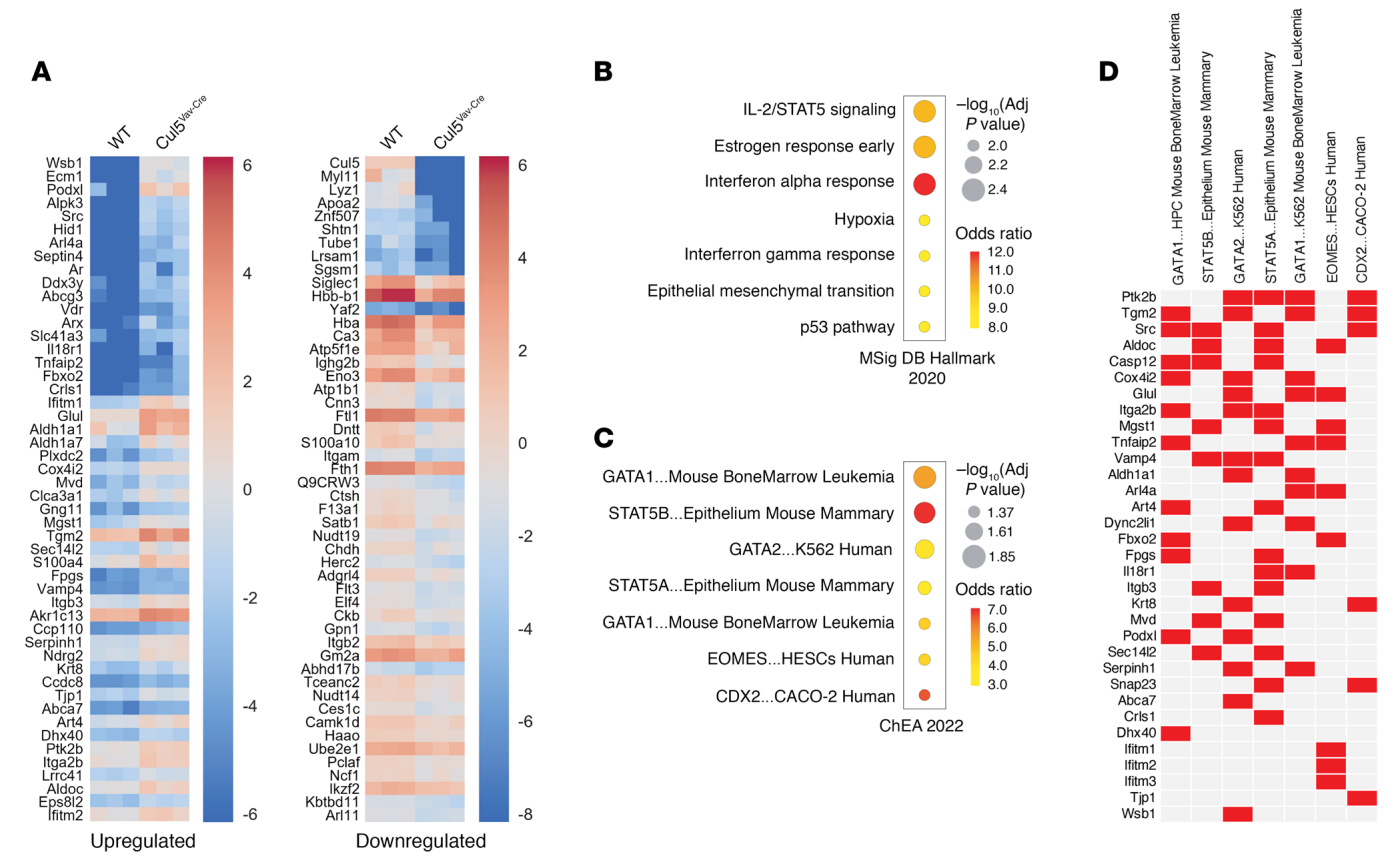
Cul5^Vav-Cre^ LSKs have increased STAT5 target protein abundance. (**A**) Top 50 significantly upregulated and downregulated proteins in Cul5^Vav-Cre^ versus WT LSKs. (**B** and **C**) Gene set enrichment analysis of proteins upregulated in Cul5^Vav-Cre^ LSKs compared with WT LSKs (log_2_ fold change > 1, *P* < 0.05) from Molecular Signatures Database (MSigDB) Hallmark collection (https://www.gsea-msigdb.org/gsea/msigdb/index.jsp) (v2020) (**B**) and ChIP Enrichment Analysis database (v2022) (**C**). (**D**) Overlap of significantly upregulated genes in Cul5^Vav-Cre^ LSK WCP and ChEA datasets. Male and female mice of the following ages were analyzed: (**A**–**D**) 8–14 weeks. (*n* = 3 biological replicates.)

To look more broadly at differences between WT and Cul5^Vav-Cre^ HSPCs, we performed gene set enrichment analysis on WCP of sorted LSK cells from bone marrow. We assessed the differential regulation of proteins involved in cell signaling with a *P* value less than 0.05 and greater than 2-fold change in Cul5^Vav-Cre^ compared with WT LSKs. We plotted the top 50 upregulated and downregulated proteins in Cul5^Vav-Cre^ LSKs to assess protein-specific differences compared with WT LSKs (Figure 6A). We then used Enrichr (51–53) to assess the overlap of upregulated proteins in Cul5^Vav-Cre^ LSKs that are enriched in immune pathways with the Molecular Signatures Database (MSigDB) Hallmark collection (https://www.gsea-msigdb.org/gsea/msigdb/index.jsp) and transcription factor binding with the ChIP Enrichment Analysis database. The top 7 statistically significant overlapping immune gene signatures in unstimulated cells included IL-2/STAT5 signaling, estrogen response early, interferon gamma response, interferon alpha response, and p53 pathway (Figure 6B). STAT5A and STAT5B target genes from mouse mammary epithelium and GATA1 and GATA2 target genes from mouse bone marrow leukemia datasets were upregulated in Cul5^Vav-Cre^ LSK WCPs (Figure 6, C and D). These results highlight the overrepresentation of proteins affiliated with STAT5 signaling, HSCs, and potential emergency hematopoiesis targets in Cul5^Vav-Cre^ mice.

### JAK1/2 inhibition normalizes hematopoiesis in Cul5^Vav-Cre^ mice.

**Figure 7:**
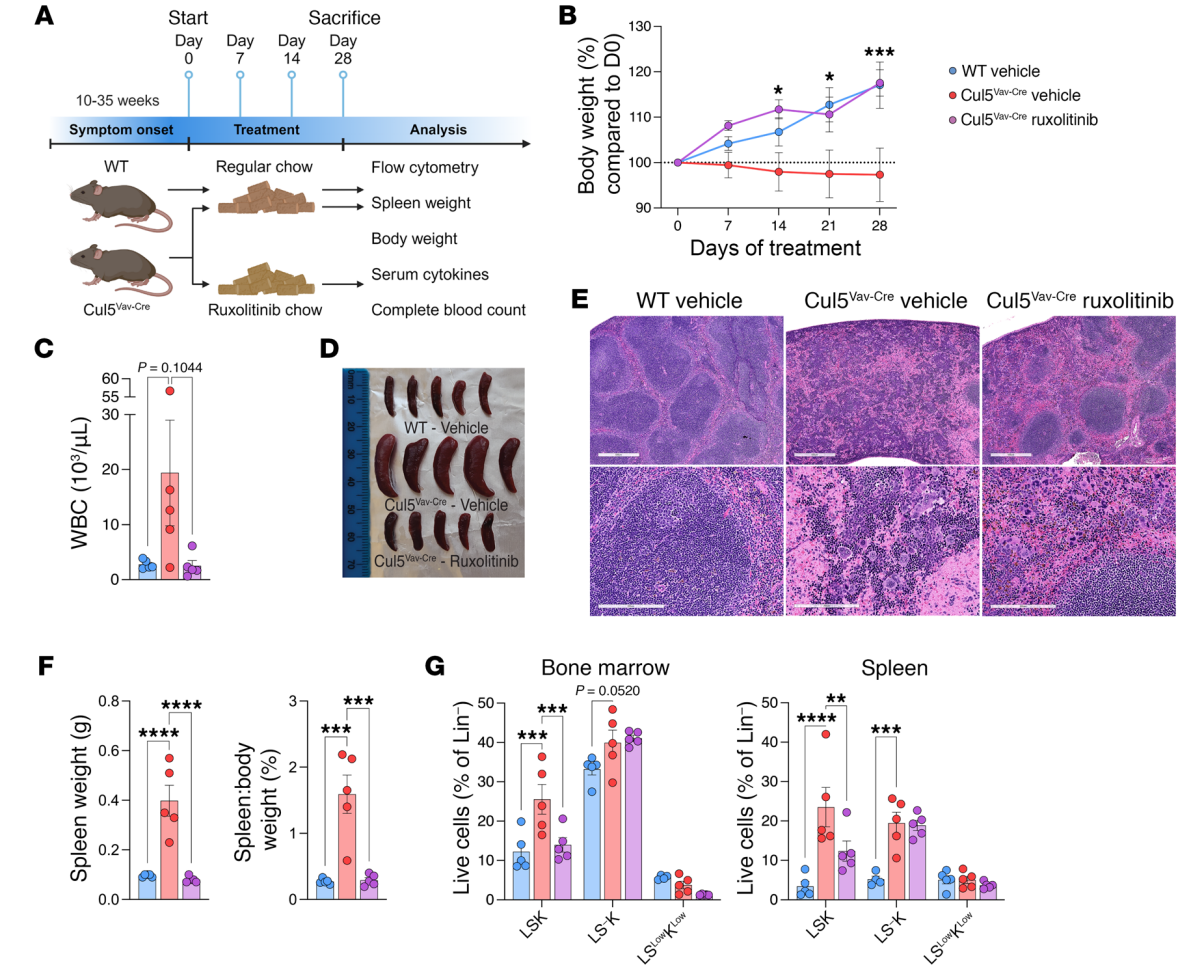
Ruxolitinib treatment normalizes hematopoiesis in Cul5^Vav-Cre^ mice. (**A**) Workflow of in vivo ruxolitinib treatment. (**B**) Change in body weight percentage of WT and Cul5^Vav-Cre^ vehicle-treated and Cul5^Vav-Cre^ ruxolitinib-treated mice. Asterisks denote significant difference between Cul5^Vav-Cre^ vehicle and Cul5^Vav-Cre^ ruxolitinib groups. (**C**) White blood cell (WBC) count in WT and Cul5^Vav-Cre^ vehicle- and Cul5^Vav-Cre^ ruxolitinib-treated whole blood. (**D**) Spleens of WT and Cul5^Vav-Cre^ vehicle- and Cul5^Vav-Cre^ ruxolitinib-treated mice. (**E**) Representative H&E of spleens from WT and Cul5^Vav-Cre^ vehicle- and Cul5^Vav-Cre^ ruxolitinib-treated mice. Scale bars: 500 µm (top), 200 µm (bottom). (**F**) Spleen weight and spleen/body weight ratios in WT and Cul5^Vav-Cre^ vehicle- and Cul5^Vav-Cre^ ruxolitinib-treated mice. (**G**) Percentage of lineage-negative populations in WT and Cul5^Vav-Cre^ vehicle- and Cul5^Vav-Cre^ ruxolitinib-treated bone marrow and spleen. The following tests were used to determine significance: (**B**) unpaired *t* test with Holm-Šidák correction; (**C**, **F**, and **G**) 2-way ANOVA with Holm-Šidák correction. *P < 0.05; **P < 0.01; ***P < 0.001; ****P < 0.0001. Male and female mice of the following ages were analyzed: (**A**–**G**) 10–30 weeks. (*n* = 5 per group.)

Ruxolitinib is a JAK1/2 inhibitor used to treat patients with myeloproliferative disorders as well as acute and chronic graft-versus-host disease after stem cell transplants. If the phenotypic alterations seen in Cul5^Vav-Cre^ mice were primarily due to elevated p-STAT5 signaling, we reasoned that treatment of these mice with ruxolitinib to reduce p-STAT5 signaling should alleviate these symptoms. We fed one group of Cul5^Vav-Cre^ mice with control chow and another with ruxolitinib chow for 28 days. We compared these mice with WT mice fed with control chow (Figure 7A). While Cul5^Vav-Cre^ mice were unable to maintain normal body weight, ruxolitinib-treated Cul5^Vav-Cre^ mice gained weight at a rate similar to that of WT controls (Figure 7B). Treated Cul5^Vav-Cre^ mice also had normalized white blood cell numbers (Figure 7C) and TPO serum levels (Supplemental Figure 6A), but unchanged red blood cells, hematocrit, hemoglobin, and platelets (Supplemental Figure 6B). When analyzed at the end of the 28 weeks, ruxolitinib-treated Cul5^Vav-Cre^ mouse spleen weights were similar to those of WT controls (Figure 7, D and F), and splenic architecture improved in comparison with Cul5^Vav-Cre^ mice that were fed control chow (Figure 7E). Treatment of Cul5^Vav-Cre^ mice normalized LSKs in both bone marrow and spleen in comparison with untreated Cul5^Vav-Cre^ mice (Figure 7G and Supplemental Figure 6D). In addition, treated mice had reduced myeloid populations in spleen and lymph nodes (Supplemental Figure 6C), and a restoration of B cells in spleen and bone marrow (Supplemental Figure 6E). Cul5^Vav-Cre^ mice demonstrated a robust response to JAK1/2 inhibition that normalizes hematopoiesis, indicated by reversal of splenomegaly, reduction of extramedullary hematopoiesis, and improved myeloid-lymphoid balanced differentiation.

## Discussion

Modulation of JAK/STAT signaling by SOCS proteins and E3s allows cells to rapidly respond and adapt to environmental cues to maintain homeostasis or expand and differentiate during stress or infection. Dysregulation of JAK/STAT signaling can lead to malignant transformation, including the development of myeloproliferative neoplasms, acute myeloid leukemia (54–57), and T cell acute lymphoblastic leukemia (58). For these reasons, it is imperative to investigate the mechanisms of JAK/STAT regulation in HSCs to provide insight into normal function and leukemia development. STAT5 is a promising clinical target because of its function in regulating proliferation of cancer stem cells (59–61). Small-molecule inhibitors of STAT5 are being developed for treatment of myeloproliferative disorders and leukemias (62), as well as solid cancers (63). Global inhibition of STAT5 may result in off-target effects, due to the varied functions and targets of STAT5 in different cell types (64).

Targeted protein degradation, using drugs like lenalidomide (65), has been an effective chemotherapeutic intervention. Attempts are under way to harness this technology using precision medicine. There is particular interest in designing proteolysis-targeting chimeras (PROTACs) that utilize engineered E3 ubiquitin ligases for targeted degradation of proteins previously considered “undruggable” (66). Even when proteins are targetable by small-molecule inhibition, patients often have low response rates or develop resistance to them (67). Few E3s are currently being utilized as PROTACs, even though the human genome encodes over 600 putative E3 enzymes. One successful PROTAC with CUL5 substrate receptors ASB1 and SOCS2 was designed to target a modified GFP substrate (68, 69), demonstrating that CUL5-based PROTACs can be successfully utilized to target proteins of interest. Our research demonstrates that CUL5 binds 9 different substrate receptors in cultured HSPCs, broadening the repertoire of PROTAC designs that are feasible and relevant for HSCs and myeloproliferative neoplasm. There are currently 10 clinical trials for PROTACs, and many more are in development for hematological diseases, including myeloproliferative neoplasm, and other cancers (70, 71). Better understanding how CUL5 functions in various cell types and in response to different stimuli will aid in the design of new PROTACs.

The function of CUL5 in HIV and solid cancers has been studied extensively, but its role in immune cell function is largely assumed to be due to its interaction with SOCS proteins. SOCS proteins canonically regulate JAK/STAT signaling by working with E3 complexes to degrade cytokine receptors or JAK, by blocking the p-STAT5 docking site on cytokine receptors, or by inhibiting JAK2 through a kinase inhibitory region (14). p-STAT5 itself is ubiquitinated and degraded by c-Cbl in response to growth hormone stimulation (72), but the E3 responsible for this function downstream of IL-3 stimulation has not been identified (44). Neither loss of SOCS1, SOCS2, nor SOCS3 (18, 19, 73, 74) recapitulates the phenotype displayed by loss of CUL5 in our system, demonstrating a function of CUL5 that is in part independent of those substrate receptors.

We also identified convergent proteins that are bound by CUL5, LRRC41, and STAT5 in bone marrow cells and are also upregulated in Cul5^Vav-Cre^ LSKs, providing an unexpected insight into further mechanistic discovery of CUL5 function in HSCs. Pathway analysis of WCP identified enrichment of estrogen, IFNA/G, p53, and GATA1/2 signaling in Cul5^Vav-Cre^ LSKs over WT. Estrogen can induce STAT5 expression in mammary cells (75). Both IFNA and IFNG play roles in HSC proliferation and function during inflammatory events (76, 77). p53 has been shown to regulate HSC quiescence and cell fate decisions (78, 79), and also shares target genes with STAT5 (80). GATA1 is required for megakaryocyte and platelet differentiation (81), while GATA2 is required for HSC survival (82, 83). The relationship between STAT5 and GATA1/2 has also been established in mast cells and basophils (84), HSCs (85), and erythroid cells (86). The overlap in STAT5 with these various pathways may provide further evidence for the importance of CUL5 in regulating STAT5 activity.

Increased IL-3 signaling has been demonstrated to downregulate CXCR4 expression (87), which is required for HSC bone marrow homing (41), maintenance of HSC quiescence (88), and B cell development (89). IL-3 has been shown to drive emergency hematopoiesis during sepsis (8) and to negatively impact the repopulation capacity of HSCs (9, 10). A recent study published on the function of CUL5 found that excessive megakaryocyte differentiation and HSC bias were independent of TPO and IFNA/B signaling, but dependent on IL-3 receptor function (32). Studies using a mouse model with a mutant STAT5 that is unable to become tyrosine-phosphorylated demonstrated the function of p-STAT5 in activation and differentiation of HSCs (90). The correlation between these studies and the phenotypes we described in Cul5^Vav-Cre^ mice suggests that CUL5 limits IL-3 signaling in HSCs to promote balance of self-renewal versus differentiation and lineage bias in hematopoiesis.

There are a few limitations to the techniques that we used for these studies. First, we performed immunoprecipitation of CUL5 on a heterogeneous population of cells that included progenitors, but also other immature myeloid populations. While this was the most technically feasible method using primary cells, it leaves open the possibility that some of the substrate receptors we found bound by CUL5 are not relevant to HSC function. The flow cytometry analysis we performed allowed us to assess the contribution of IL-3, SCF, and TPO individually to substrate receptor expression. But for some substrate receptors, like WSB1, we were unable to detect the same differences in abundance in WT versus Cul5^Vav-Cre^ LSKs in the flow cytometry analysis that we observed in the WCP analysis. This disparity could be due to low sensitivity of the antibody that we used for analysis compared with the high sensitivity of mass spectrometry analysis. Finally, we were unable to definitively identify p-STAT5 as the substrate of the CRL5 complex with LRRC41. While it is still possible that STAT5 is directly ubiquitinated by CRL5, it is also possible that the increase in STAT5 signaling is an indirect consequence of the function of CUL5. Despite these open questions that require clarification in future studies, we have established a clear link between CUL5 loss, an emergency hematopoiesis–like or myeloproliferative-like phenotype, and IL-3–dependent p-STAT5 signaling.

Collectively, our study identifies 9 different substrate receptors that bind CUL5 in bone marrow cells, as well as STAT5, downstream of TPO, SCF, and IL-3 stimulation. Proteomic analysis further defined LRRC41 and WSB1 as the most abundant substrate receptors in WT LSKs, two proteins that were also highly upregulated in Cul5^Vav-Cre^ LSKs. Furthermore, we identified an increase in LRRC41 levels following IL-3 stimulation in both WT and Cul5^Vav-Cre^ HSCs. Our data suggest that CUL5 forms a complex with LRRC41, and CUL5 facilitates inhibition of p-STAT5 to control HSPC proliferation and cell fate. These findings elucidate an important role for CUL5 in HSPC function and identify substrate receptor interactions that may facilitate designing new therapies for hematological diseases.

## Methods

Further information can be found in Supplemental Methods.

### Sex as a biological variable.

Our study examined male and female animals, and similar findings are reported for both sexes.

### Animals.

Cul5^fl/fl^ mice were generated using homologous recombination as described previously (33); these services were provided by Taconic Labs. Vav-iCre mice (B6.Cg-Commd10^Tg(Vav1-icre)A2Kio^/J) were purchased from The Jackson Laboratory. All mice were bred in-house under specific pathogen–free conditions in the animal facility at the Children’s Hospital of Philadelphia (CHOP). Mice were housed at 18°C–23°C and 40%–60% humidity with 12-hour light/12-hour dark cycles. Mice used for these studies were aged 5–55 weeks and were matched for each experiment.

### Antibodies and reagents.

Reagents and antibodies used in these studies are listed in Supplemental Tables 1 and 2.

### Bone marrow chimeras.

Recipient mice (CD45.1×CD45.2) were lethally irradiated (550 + 550 cGy separated by 4 hours) using an X-Rad Irradiator. For whole bone marrow competitive transplants, cells from control (CD45.1) and Cul5^Vav-Cre^ (CD45.2) mice were isolated, T cell depleted by magnetic-activated cell sorting (MACS) (Miltenyi), resuspended in PBS, and mixed at a 1:1 ratio. Recipients were injected with 1 × 10^6^ cells by retro-orbital injection. Mice were euthanized and analyzed 5–9 weeks after transplant.

For sorted competitive transplants, HSCs (LSK CD150^+^ CD48^–^) and MPPs (LSK CD150^–^ CD48^–/+^) were sorted from control (CD45.1) and Cul5^Vav-Cre^ (CD45.2) bone marrow. Recipients were injected with 100 WT and 100 Cul5^Vav-Cre^ HSCs or MPPs with 500,000 CD45.1×CD45.2 whole bone marrow cells by retro-orbital injection. Peripheral blood chimerism was assessed every 4 weeks by submandibular bleed followed by flow cytometry. Complete blood counts (CBCs) were analyzed at 12, 16, and 20 weeks. Mice were euthanized and analyzed 20 weeks after transplant.

For competitive serial transplants, cells from control (CD45.1) and Cul5^Vav-Cre^ (CD45.2) mice were isolated, resuspended in PBS, and mixed at a 10:1 ratio. Primary recipients were injected with 2 × 10^6^ cells by retro-orbital injection. Bone marrow from primary recipient mice was isolated at 15 weeks after transplant. Secondary recipients were injected with 5 × 10^6^ whole bone marrow cells from primary recipients by retro-orbital injection.

All transplant recipients were maintained on Sulfatrim antibiotic water (Pharmaceutical Associates, Inc.) for the first 4 weeks after transplant, and body weights were recorded once a week.

### BrdU.

Mice were supplied with BrdU in drinking water (0.8 mg/mL) for 6 days. Bone marrow cells were isolated and lineage-negative cells were enriched by MACS (Direct Lineage Depletion Kit, Miltenyi) per the manufacturer’s instructions. Isolated cells were stained using the BrdU Flow Kit (BD Biosciences) following the manufacturer’s instructions. Samples were acquired on the Cytek Aurora.

### Complete blood count.

Blood samples were collected by cardiac puncture and stored in EDTA tubes at room temperature (<4 hours) or at 4°C (>4 hours) until analysis on the Sysmex XT-2000iV Automated Hematology Analyzer.

### CFU assays.

Sorted WT and Cul5^Vav-Cre^ LSK and LS^–^K spleen cells were plated in MethoCult M3434 and incubated at 37°C per the manufacturer’s instructions (Stem Cell Technologies). Colonies were identified and enumerated on day 8–11 by hand on an inverted microscope. For CFU assays with inhibitors, Cul5^Vav-Cre^ spleen cells were plated in MethoCult M3434 with ruxolitinib, fedratinib, or binimetinib (1 μM) for 11 days. Colonies were dissociated in IMDM using a pipette, and then live single cells were counted with trypan blue on a hemocytometer.

### Flow cytometry and sorting.

Single cells were stained with LIVE/DEAD Blue (Invitrogen) for 10 minutes in PBS at room temperature. For flow cytometry, cells were incubated with antibodies in FACS buffer for 30 minutes on ice, washed with FACS buffer, fixed with BD Cytofix/Cytoperm, and acquired on the Cytek Aurora. FlowJo software (BD Life Sciences) was used for analysis. For sorting, lineage-negative cells were enriched by MACS (Direct Lineage Depletion Kit, Miltenyi) per the manufacturer’s instructions. Lineage-negative cells were then incubated with antibodies in MACS buffer for 30 minutes on ice, washed, and resuspended in MACS buffer and stored on ice. LSKs (whole-cell proteome and CFU), LS^−^Ks (CFU), CD34^lo^ CD150^+^ CD48^−^ LSKs (HSC culture), CD150^+^ CD48^−^ LSKs (bone marrow transplant), and CD150^–^ CD48^−/+^ LSKs (bone marrow transplant) were sorted on the Cytek Aurora CS.

### HSC culture.

CD34^lo^ HSCs were cultured as previously described (43). Briefly, sorted cells were plated in fibronectin-coated 96-well plates in F-12 medium with polyvinyl alcohol, penicillin/streptomycin, HEPES, insulin-transferrin-selenium-ethanolamine, TPO (100 ng/mL), SCF (10 ng/mL), and IL-3 (10 ng/mL). Medium was changed every 2–3 days, and cells were passaged at 90% confluence. After 19–28 days, cells were incubated with bortezomib (50 nM) for 2 hours, collected, washed in PBS, and frozen as pellets at –80°C for immunoprecipitation.

### Immunoprecipitation.

Cells were lysed for 30 minutes on ice in 1 mL per 1 × 10^8^ cells of lysis buffer (nuclease-free H_2_O, 1% NP-40, 100 mM NaCl, 50 mM Tris-HCl, 2× cOmpleteMini, EDTA-free Protease Inhibitor Cocktail (Roche), 2× Halt protease and phosphatase inhibitor (ThermoFisher), 100 μM PR-619, 2× o-Phenanthroline) and centrifuged at 20,000*g* for 10 minutes to clarify lysate. For CUL5 immunoprecipitation, cell lysates were incubated with IgG antibody for 20 minutes at room temperature, followed by incubation for 2 hours with Dynabeads (Invitrogen) washed with PBS with 0.1% Tween-20. IgG beads were removed using a magnet. Lysates were then incubated with CUL5 antibody (4 μg/mg) or rabbit IgG antibody overnight at 4°C. Washed Dynabeads were added to lysates and incubated overnight at 4°C. CUL5- and IgG-bound beads were then removed from the lysate on a magnet and washed 3 times with PBS with 0.1% Tween-20. Beads were centrifuged and stored as pellets at –80°C until preparation for mass spectrometry. For STAT5 and LRRC41 immunoprecipitation, IL-3–stimulated (20 ng/mL) cells were washed 3 times with cold PBS. Cells were resuspended in 3 disuccinimidyl dibutyric urea (1 mM) and incubated for 10 minutes at room temperature. The cross-linker was quenched by addition of Tris (20 mM) for 5 minutes at 4°C. Pellets were washed with PBS and lysed. Cell lysates were incubated with IgG-bound Dynabeads washed with lysis buffer at 4°C for 1 hour. IgG beads were removed using a magnet. Lysates were then incubated with STAT5 antibody (10 μL/mg), LRRC41 antibody (4 μg/mg), or rabbit IgG antibody overnight at 4°C. Washed Dynabeads were added to lysates and incubated for 4 hours at 4°C. Protein-bound Dynabeads were then removed from the lysate on a magnet and washed 3 times with lysis buffer. Beads were centrifuged and stored as pellets at –80°C until preparation for mass spectrometry.

### In vivo ruxolitinib.

Ruxolitinib was prepared in Nutra-Gel (2 g/kg) (Bio-Serv) per the manufacturer’s instructions. Mice were fed ruxolitinib or control chow ad libitum (8 g/d) for 4 weeks. Body weights were recorded once a week. On day 28, mice were euthanized, and blood was collected by cardiac puncture for CBC analysis. Spleens, lymph nodes, and femora were collected, stained, and analyzed by flow cytometry and/or histology.

### Phospho-flow.

Isolated bone marrow cells were stained with LIVE/DEAD Blue for 10 minutes at room temperature followed by surface markers in IMDM with 2% FBS at 37°C for 30 minutes. Cells were washed with IMDM with 2% FBS and centrifuged at 400*g* for 5 minutes at 4°C, then incubated in IL-3, TPO, or SCF (20 ng/mL; 10^7^ cells/1 mL) in IMDM with 10% FBS at 37°C. At indicated time points, cells were immediately fixed with 1.5% paraformaldehyde at room temperature for 10 minutes. Cells were washed in PBS with 2% FBS, permeabilized in ice-cold methanol, and stored at –80°C until intracellular staining. Cells were washed in PBS with 2% FBS and centrifuged for 400*g* for 5 minutes at 4°C, then incubated with primary antibodies for 30 minutes on ice. Cells were washed in PBS with 2% FBS and centrifuged for 400*g* for 5 minutes at 4°C and incubated with secondary antibody for 30 minutes on ice. Samples were washed in PBS with 2% FBS and centrifuged for 400*g* for 5 minutes at 4°C, resuspended in PBS with 2% FBS, then acquired on the Cytek Aurora on the same day. For bortezomib experiments, cells were incubated with 500 nM bortezomib (1 mL/10^7^ cells) for 30 minutes before stimulation with IL-3 (50 ng/mL) for the indicated times.

### Serum cytokines.

Blood was collected by cardiac puncture, and serum was isolated by centrifugation in serum tubes. Serum was stored at –80°C and thawed at room temperature upon use. Cytokine levels were analyzed with LEGENDplex Mouse HSC Panel (BioLegend) per the manufacturer’s instructions. Samples were acquired on the Cytek Aurora. LEGENDplex Data Analysis Software Suite (BioLegend) was used to calculate cytokine concentrations. Analytes with less than 50 beads captured were excluded from analysis.

### Stem cell homing assay.

Recipient mice (CD45.1) were lethally irradiated (650 + 650 cGy separated by 4 hours) using an X-Rad Irradiator. Bone marrow cells from WT (CD45.1×CD45.2) and Cul5^Vav-Cre^ (CD45.2) mice were isolated and lineage-depleted by MACS. The proportion of HSCs from each donor was calculated by flow cytometry. Total lineage-negative cells were normalized to achieve a 1:1 ratio of HSCs. Cells were resuspended in PBS and injected by retro-orbital injection into irradiated recipients (CD45.1). After 15 hours, bone marrow was isolated from recipients, lineage-depleted, and stained. The presence of stem cells from each donor was determined by flow cytometry.

### Whole-cell proteomics analysis.

Sorted LSK cells from bone marrow for unstimulated samples were washed twice with PBS and stored as pellets at –80°C. Stimulated samples were incubated with IL-3 (10 ng/mL), SCF (10 ng/mL), and TPO (100 ng/mL) with bortezomib (50 nM) in IMDM with 10% FBS for 1 hour. Cells were washed twice with PBS and stored as pellets at –80°C.

Whole-cell proteomics data were quantile-normalized and analyzed for differential abundance. Differentially abundant proteins were identified by the performing of Student’s *t* tests on the normalized data (α = 0.05). Differentially abundant proteins were analyzed for enrichment using the MSigDB Hallmark Genes dataset (v2020) and the ChIP Enrichment Analysis (ChEA) dataset (v2022) and sorted by Bonferroni-corrected *P* values for visualization. Top terms and associated adjusted *P* values for MSigDB Hallmark genes and ChEA genes were found using the Enrichr API function in the gseapy package (v1.0.4) in Python (https://www.python.org/). Visualization of gene ontology was performed using modified functions from the gseapy package in Python.

Further information about proteomics methods is included in Supplemental Methods.

### Statistics.

The following methods were used to calculate significance: unpaired *t* tests (Figure 1, A and B, Figure 2, C and E, Figure 3B, Figure 4D, Supplemental Figure 2, C and E, and Supplemental Figure 4C), unpaired *t* test with Holm-Šidák correction (Figure 1, D–G, Figure 2, B, D, and G, Figure 3, A, C, E, and G, Figure 4, B, C, and F, Figure 5, E, H, and I, Supplemental Figure 1, A, B, E, and F, Supplemental Figure 2, B, D, and F, Supplemental Figure 3, A–D, Supplemental Figure 4, A, B, E, and F, and Supplemental Figure 5A), paired *t* test (Figure 3B), 1-way ANOVA with Holm-Šidák correction (Figure 3H, Figure 7, C and F, and Supplemental Figure 6, A and B), 2-way ANOVA with Holm-Šidák correction (Figure 5F, Figure 7, B and G, and Supplemental Figure 6, C–E), and Spearman’s correlation (Supplemental Figure 4, C and D). All *t* tests performed were 2-tailed. Outliers were excluded from flow cytometry and CBC data in Figures 1, 2, and 7 and Supplemental Figures 1, 2, and 6 using the ROUT method (*Q* = 0.1%). *P < 0.05; **P < 0.01; ***P < 0.001; ****P < 0.0001. Data represent mean ± SEM.

### Study approval.

Animal housing, care, and experimental procedures were approved by and performed in compliance with the Institutional Animal Care and Use Committee at the Children’s Hospital of Philadelphia (https://www.research.chop.edu/institutional-animal-care-and-use-committee).

### Data availability.

The mass spectrometry proteomics data were deposited to ProteomeXchange (91) via the PRIDE partner repository (92) with the dataset identifier PXD046958. Values for all data points in graphs are reported in the Supporting Data Values file.

## Author contributions

SAT, DDK, NP, AAD, IG, YOB, LAS, JR, and HF performed and assisted with experiments. SAT and DDK analyzed results and made figures. CST assisted with public database searching and reviewed the manuscript. RLB provided expertise on bone marrow transplant experimental design and interpretation, and provided editing of and feedback on the manuscript. SAT and PMO designed experiments and prepared the manuscript.

## Supplementary Material

Supplemental data

Supporting data values

## Version 1 06/26/2025

In-Press Preview

## Version 2 09/02/2025

Electronic publication
