# Supplementary material for: The E3 ubiquitin ligase Cul5 regulates hematopoietic stem cell function for steady-state hematopoiesis in mice
Source: J Clin Invest. 2025 Jun 26;135(17):e180913. doi: 10.1172/JCI180913 (PMC12404750; doi:10.1172/JCI180913)
Supplement: Supplemental data [file jci-135-180913-s084.pdf]

Supplemental Data:

The E3 ubiquitin ligase Cul5 regulates hematopoietic stem cell function for steady-state hematopoiesis

Siera A Tomishima[1], Dale D Kim[1], Nadia Porter[2], Ipsita Guha[2], Asif A Dar[2], Yohaniz Ortega-Burgos[1], Jennifer Roof[3], Hossein Fazelinia[3], Lynn A Spruce[3], Christopher S Thom[4,5] Robert L Bowman[6] and Paula M Oliver[1,2,*]

Supplementary Materials and Methods

Bone Marrow Preparation

Bone marrow centrifugation tubes were prepared by cutting the bottom of a 0.5 mL microcentrifuge tube with a razor blade and placing it inside of a 2 mL microcentrifuge tube. Femur, tibia, humerus, pelvis, radius and ulna were isolated and placed in a petri dish with PBS then cleaned of all muscle by scraping with a razor blade. Bones were cut in half using a razor blade and placed cut side down into the prepared centrifugation tubes. Bones were centrifuged for 1 minute at 20,000×g in a microcentrifuge at room temperature. The 0.5 mL microcentrifuge tubes containing bones were removed and discarded, and the pelleted bone marrow cells were resuspended in 1 mL of RPMI with 1% FBS and transferred to a conical. The 2 mL microcentrifuge tube was washed with 1 mL of RPMI with 1% FBS and transferred to a conical and centrifuged for 5 minutes at 300×g at 4°C. Red blood cells were lysed in 5 mL of ACK buffer for 5 minutes at room temperature, then neutralized with 20 mL of PBS and centrifuged for 5 minutes at 300×g at 4°C. Cells were resuspended in RPMI with 1% FBS and transferred to a new conical through a new 70 μM cell strainer. Live cells were counted on a hemocytometer with trypan blue.

Histology

CD45R (BD Pharmingen, 550286), CD3 (Abcam, 215212), and CD41 (Abcam, ab134131) antibodies were used to stain formalin fixed paraffin embedded tissue slides. Staining was performed on a Bond Rx automated staining system (Leica Biosystems). The Bond Intense R (Leica Biosystems DS9263, CD45R) and Refine (Leica Biosystems DS9800, CD3 and CD41) staining kits were used. The standard protocols were followed with the exception of the primary antibody incubation which was extended to 1 hour at room temperature and the post primary step was excluded for the Refine staining kit. Antibodies were used at 1:50 (CD45R) and 1:100 (CD3, CD41) dilutions and antigen retrieval was performed with E1 (Leica Biosystems, AR9961) retrieval solution for 20 minutes. Slides were rinsed, dehydrated thru a series of ascending

concentrations of ethanol and xylene, then coverslipped. Stained slides were then digitally scanned at 20× magnification on an Aperio CS-O slide scanner (Leica Biosystems).

Spleen and Lymph Node Preparation

Isolated spleens and lymph nodes were placed in a petri dish with 5 mL of RPMI with 1% FBS on a 70 μM cell strainer and mashed with the plunger of a 1 mL syringe. The single cell suspensions were transferred to a conical, then the strainers and dishes were washed two times with 5 mL of RPMI with 1% FBS and added to the conical. The cells were centrifuged for 5 minutes at 300×g at room temperature. For spleen preparation, red blood cells were lysed in 2 mL of ACK lysing buffer for 2 minutes at room temperature, then neutralized with 8 mL of PBS and centrifuged for 5 minutes at 300×g at 4°C. Cells were resuspended in RPMI with 1% FBS and transferred to a new conical through a new 70 μM cell strainer. Live cells were counted on a hemocytometer with trypan blue.

Proteomics Methods

Protein Extraction and Estimation

Samples were solubilized in 50 μL of proprietary Lyse buffer (PreOmics) containing SDC, reducing agent, and alkylating agent (Kulak, et al 2014). They were then heated to 90°C for 10 minutes to lyse cells or elute immunoprecipitated protein off of the beads as well as reduce and alkylate proteins. Samples were then centrifuged at 20,000×g for 10 minutes to clarify lysate. Protein yield was determined via intrinsic tryptophan fluorescence on a Take3 Trio microvolume plate read in a Synergy H1 Multimode plate reader (Agilent, Santa Clara, CA). Fluorescence values were plotted on a reference curve of serially diluted in-house generated E.coli lysate.

In-Solution Digestion

Samples were digested with the PreOmics iST Sample Preparation kit, per the manufacturer's protocol (Kulak, et al 2014). Briefly, samples were transferred to the PreOmics cartridge, and 50 μL of digest buffer containing trypsin protease was added. Samples were incubated for 1.5 hours at 37°C before the reaction was stopped. Peptides were washed twice and eluted from

the cartridge using provided buffers. Eluates were then dried by vacuum centrifugation and reconstituted in 0.1% TFA containing iRT peptides (Biognosys, Schlieren, Switzerland). IP samples were then analyzed. Whole proteome samples were loaded onto Evotip disposable C18 trap columns (Evosep, Denmark) per manufacturer's protocol. Briefly, Evotips were rinsed with 0.1% formic acid in acetonitrile, activated by soaking in 1-propanol, and washed with 0.1% formic acid. Sample was applied and washed with 0.1% formic acid. The Evotips were kept wet with 0.1% formic acid prior to injection.

Mass Spectrometry Data Acquisition

Whole proteome samples were randomized and analyzed on an TimsTOF Pro2 mass spectrometer (Bruker Daltonics, Bremen, Germany) coupled with an Evosep One nano-LC system (Evosep) in data independent acquisition (DIA) mode. Peptides pre-loaded onto an Evotip were separated using the Evosep 30SPD method. Mobile phase A consisted of 0.1% formic acid and mobile phase B of 0.1% formic acid in acetonitrile.

For data independent acquisition with PASEF (DIA-PASEF), settings were as follows: one full MS scan with a scan range of 100-1700 m/z, and TIMS settings at 1/K0 range of 0.60-1.60 V×s/cm^2, ramp and accumulation times of 100 ms, and a ramp rate of 9.43 Hz. Peak detection thresholds were set at 10 for mass and 5000 for mobility. MS/MS were collected in two groups of 16 variable isolation windows with a mass range of 400-1201 Da, a mobility range of 0.60-1.43 1/K0, and an estimated cycle time of 1.80s. First in group scans occurred at 400 Da and 0.60 1/K0 as well as 800 Da and 0.90 1/K0. Each window spanned 26 Da and 0.30 1/K0, creating 1 Da and 0.02 1/K0 overlaps respectively.

Immunoprecipitated proteins were analyzed on an Exploris 480 mass spectrometer (ThermoFisher Scientific, San Jose, CA) coupled with an Ultimate 3000 nano-UPLC system (ThermoFisher). Samples were separated by reverse phase (RP)-HPLC on a 50 cm uPAC Neo HPLC column (ThermoFisher) coupled to an EasySpray source (ThermoFisher). Mobile phase A consisted of 0.1% formic acid and mobile phase B of 0.1% formic acid/acetonitrile. Peptides

were eluted into the mass spectrometer at 300 nL/min with each RP-LC run comprising a 90 minute gradient from 3% B to 38% B. For data dependent acquisition (DDA), the mass spectrometer was set with a master scan at R=120000, with a scan range of 300-1400, and standard AGC target. Maximum injection time and dynamic exclusion were set to auto. Charge states 2-5 were included. Top 30 data dependent MSMS scans were collected at R=15000, with first mass at 120, ACG target set to standard, automatic maximum injection time, and HCD collision energy at 30.

System Suitability and Quality Control

The suitability of both the Exploris 480 and TimsTOF Pro2 instruments was monitored using QuiC software (Biognosys, Schlieren, Switzerland) for the analysis of the spiked-in iRT peptides. Meanwhile, as a measure for quality control, we injected standard E. coli protein digest before in the middle of, and after sample set using DDA mode. The collected DDA data were analyzed in MaxQuant[1] and the output was subsequently visualized using the PTXQC[2] package to track the quality of the instrumentation.

Database Searching

The DDA and DIA raw files were processed with MaxQuant (2.0.3.0) and Spectronaut 18.1, respectively (Bruderer et al, 2015; Tyanova et al, 2016). We used reference mouse proteome including 25,508 canonical and reviewed isoforms from Uniprot appended with the list of 245 common protein contaminants. Trypsin was specified as enzyme with two possible missed cleavages. Carbamidomethyl of cysteine was specified as fixed modification and protein N-terminal acetylation and oxidation of methionine were considered variable modifications. The false discovery rate limit of 1% was set for precursors, peptides and proteins identification. The rest of the search parameters were kept as default.

Bioinformatic Analysis

Proteomics data processing and statistical analysis were performed in R. The MS2 intensity values generated by Spectronaut were used to analyze the whole proteome data. The data

were log2 transformed and normalized by subtracting the median for each sample. We filtered the data to have a complete value for a protein in at least one cohort. To compare proteomics data between groups, Limma t-test was employed to identify differentially abundant proteins, and volcano plots were generated in visualize the affected proteins while comparing different groups. Lists of differentially abundant proteins was sorted based on the p-value <0.05, yielding a prioritized list for downstream bioinformatic analysis.

Table 1. Antibodies

Antibody	Company	Clone/Catalog Number	Assay	Dilution
7-AAD	BD Biosciences	559925	FC	1:200
B220	BioLegend	RA3-6B2	FC	1:500
BrdU	BioLegend	3D4	FC	1:200
CD45R (B220)	BD Pharmingen	550286	IHC	1:50
CD3	Abcam	215212	IHC	1:100
CD11b	BioLegend	M1/70	FC	1:200
CD16/32	BioLegend	93	FC	1:250
CD19	BioLegend	6D5	FC	1:200
CD34	BioLegend	SA376A4	FC	1:250
CD41	Abcam	ab134131	IHC	1:100
CD41	BioLegend	MWReg30	FC	1:250
CD42d	BioLegend	1C2	FC	1:200
CD45	BioLegend	30-F11	FC	1:200
CD45.1	BioLegend	A20	FC	1:200
CD45.2	BioLegend	104	FC	1:200
CD48	BioLegend	HM48-1	FC	1:250
CD71	BioLegend	RI7217	FC	1:200
CD117 (c-kit)	BioLegend	2B8	FC	1:500
CD127	eBioscience	A7R34	FC	1:125
CD135	BD Biosciences	A2F10.1	FC	1:125

CD150 (SLAM)	BioLegend	TC15-12F12.2	FC	1:500
CD184 (CXCR4)	BioLegend	L276F12	FC	1:500
CISH	Aviva Biology Systems	ARP52798_P050	FC	1:200
Cul5	Fortis Life Sciences	A302-173A	IP	4 μg/mg
F4/80	BioLegend	BM8	FC	1:200
Gr-1	BioLegend	RB6-8C5	FC	1:250
IgG (α-Rabbit)	Invitrogen	A-11037	FC	1:2000
IgG (Normal Rabbit)	Cell Signaling Technologies	2729	IP	
LIVE/DEAD™ Blue	Invitrogen	L34962	FC	1:80
LRRC41	ProteinTech	20457-1-AP	FC, IP	1:200, 4 μg/mg
Ly6G	BioLegend	1A8	FC	1:200
PCMTD2	Aviva Biology Systems	ARP48826_P050	FC	1:200
Sca-1	BioLegend	D7	FC	1:125
SiglecF	BD Biosciences	E50-2440	FC	1:200
pSTAT5	Cell Signaling Technologies	C11C5	FC	1:200
STAT5	Cell Signaling Technologies	94205	IP	10 μL/mg
TCRβ	BioLegend	H57-597	FC	1:250
Ter119	BioLegend	TER-119	FC	1:250
WSB1	ProteinTech	11666-1-AP	FC	1:200

Table 2. Reagents

Reagent	Company	Catalog Number
AKC Lysing Buffer	Quality Biologicals	118-156-101
BD Cytofix/Cytoperm™ Kit	BD Biosciences	554714
BD Cytoperm™ Permeabilization Buffer Plus	BD Biosciences	561651
Binimetinib	MedChemExpress	HY-15202
Bortezomib	Sigma Aldrich	5043140001

BrdU	MedChemExpress	HY-15910
cOmplete™, Mini, EDTA-free Protease Inhibitor Cocktail	Roche	11836170001
Direct Lineage Depletion Kit, Mouse	Miltenyi	130-110-470
DMSO	Sigma Aldrich	D8418-500ML
DPBS	Gibco	14190144
DSBU	ThermoFisher	A35459
Dynabeads™ (Protein A)	Invitrogen	10001D
EDTA	Amresco	E177
F-12 Media	Gibco	11765054
FBS	Gibco	10437-028
Fedratinib	MedChemExpress	HY-10409
HALT™ Protease and Phosphotase Inhibitor Cocktail	ThermoFisher	78440
HEPES	Gibco	15630080
IL-3 (recombinant mouse)	PeproTech	213-13
IMDM	Gibco	12440053
ITS-X	Gibco	51500056
LEGENDplex™ Mouse HSC Panel	BioLegend	740677
LS Columns	Miltenyi	130-042-401
Methanol	Sigma Aldrich	179337-500ML
MethoCult™ M3434	Stem Cell Technologies	03434
NP-40	Sigma Aldrich	I8896
Nutra-Gel Diet™, Dry Mix Kit	Bio-Serv	F4798-KIT
Nutra-Gel Diet™	Bio-Serv	S4798-TRAY
o-Phenanthroline	Life Sensors	SI9649
Paraformaldehyde (16%)	Thermo Scientific	AA433689M
Pen/Strep	Gibco	15140122
Polyvinyl Alcohol	Sigma Aldrich	P8136-250G
PR-619	Life Sensors	SI96190
RPMI 1640	Cytiva Life Sciences	SH30096.01
Ruxolitinib	MedChemExpress	HY-50856
SCF (recombinant mouse)	PeproTech	250-03

Sulfatrim	Pharmaceutical Associates, Inc	00121-0854-16
TPO (recombinant mouse)	PeproTech	315-14

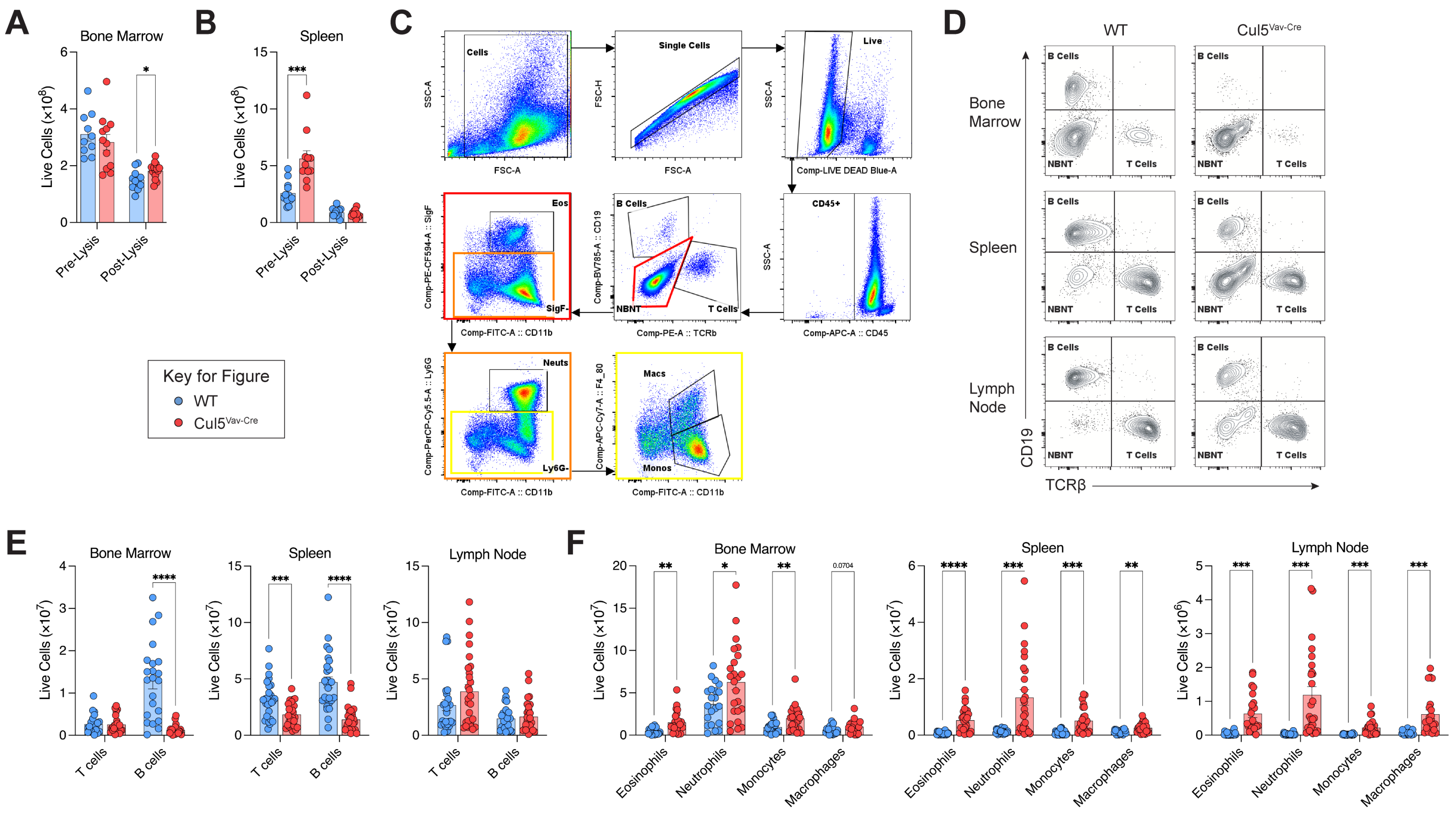

Supplemental Figure 1. Cul5$^{Vav\text{-}Cre}$ Mice Phenotype

Live cells in spleen (**A**) and bone marrow (**B**) before and after RBC lysis (n≥10). (**C**) Gating strategy for flow cytometry of mature immune cells. (**D**) Representative flow plots of B cells, T cells and non-B/non-T cells (NBNT) in bone marrow, spleen and lymph nodes from WT and Cul5$^{Vav\text{-}Cre}$ mice. Number of live B and T cells (**E**) and myeloid cells (**F**) in the spleen, bone marrow and lymph nodes (n≥21). Male and female mice, aged 5-55 weeks were analyzed. Unpaired t-tests with Holm-Šídák correction were used to determine significance. (ns <0.1 * p<0.05; ** p<0.01; *** p<0.001; **** p<0.0001)

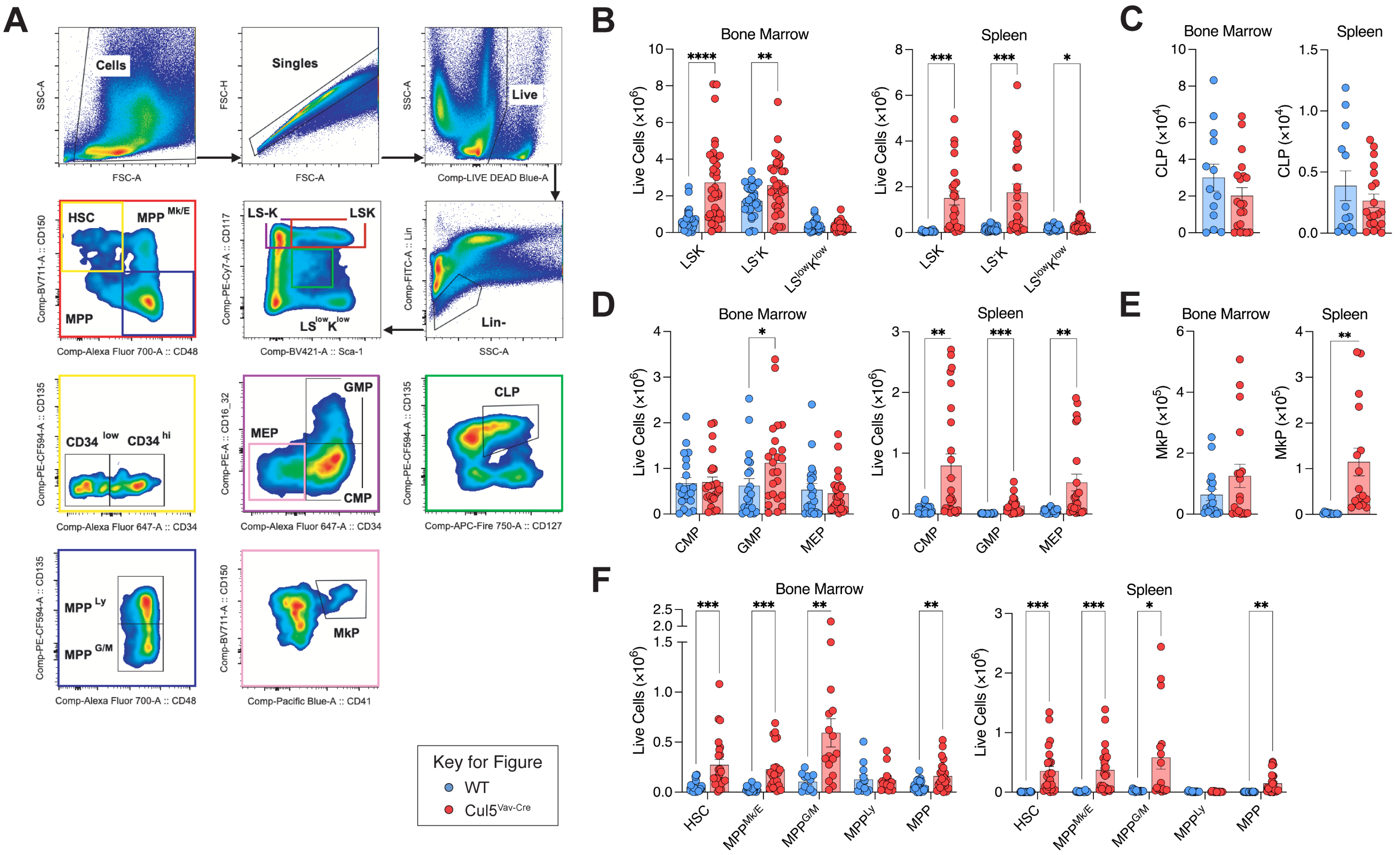

Supplemental Figure 2. HSPC Flow Cytometry

(**A**) Gating strategy for flow cytometry of HSPCs in bone marrow and spleen. Numbers of live lineage negative cells, (**B**) CLPs, (**C**) LS-K populations (**D**), MkPs (**E**) and LSK populations (**F**) in spleen and bone marrow of WT and Cul5$^{Vav\text{-}Cre}$ mice (n≥13). Male and female mice, aged 5-55 weeks were analyzed. The following tests were used to determine significance: (B, D and F) Unpaired t-test with Holm-Šídák correction; (C and E) Unpaired t-test. (ns <0.1 * p<0.05; ** p<0.01; *** p<0.001; **** p<0.0001)

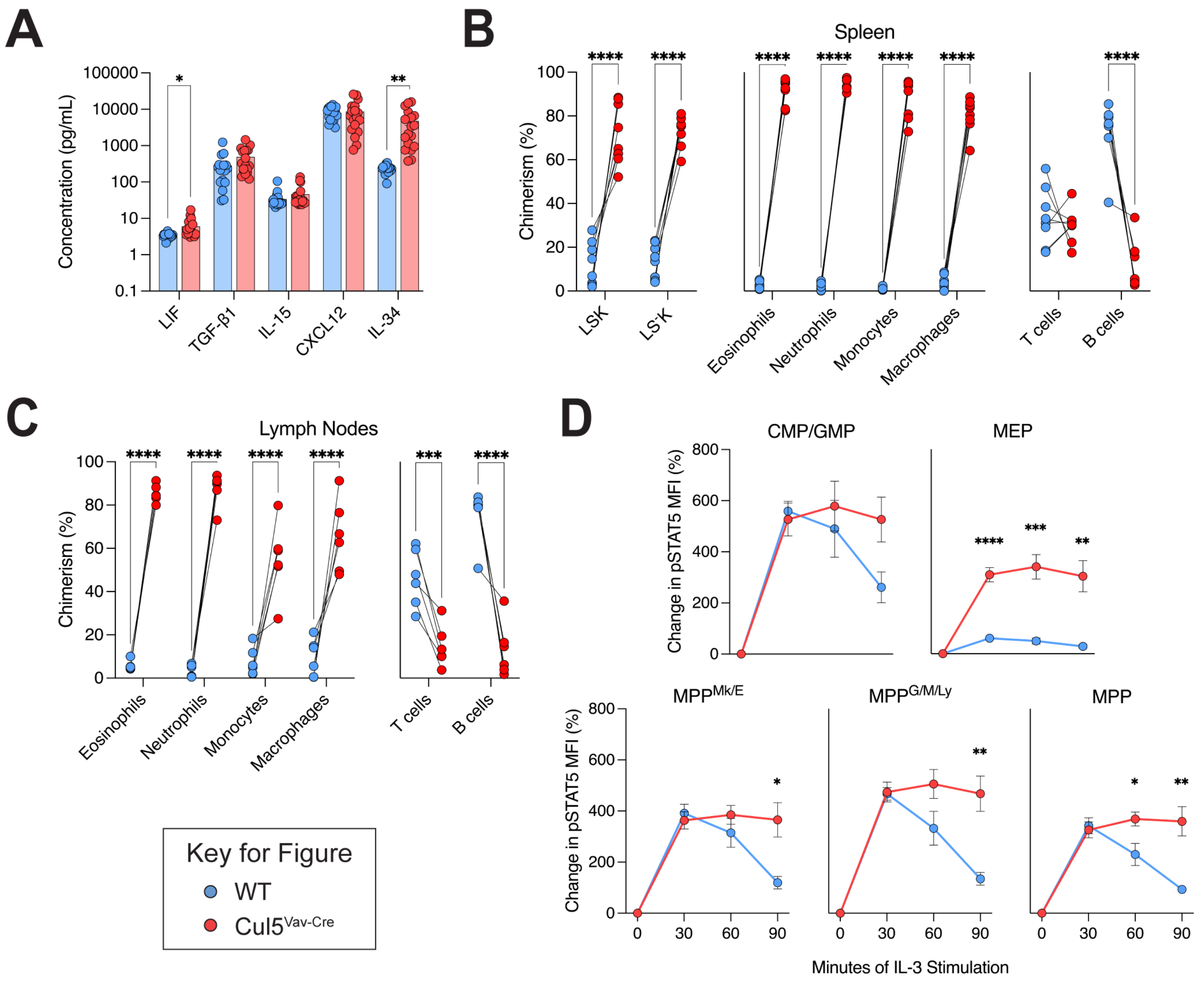

Supplemental Figure 3. Serum Cytokines, Cul5$^{Vav\text{-}Cre}$ Chimeras and PhosphoFlow

(**A**) Serum cytokine concentrations in WT and Cul5$^{Vav\text{-}Cre}$ mice (n=18). Percent chimerism of lineage negative and mature lineage populations in spleen (**B**) and lymph node (**C**) of competitive bone marrow transplants at 5-9 weeks. (n≥6). (**D**) Percent change in pSTAT5 MFI of WT and Cul5$^{Vav\text{-}Cre}$ bone marrow cells with IL-3 stimulation (n=8). Male and female mice of the following ages were analyzed: (A) 5-55 weeks; (D) 22-55 weeks. Unpaired t-tests with Holm-Šídák correction were used to determine significance. (ns <0.1 * p<0.05; ** p<0.01; *** p<0.001; **** p<0.0001)

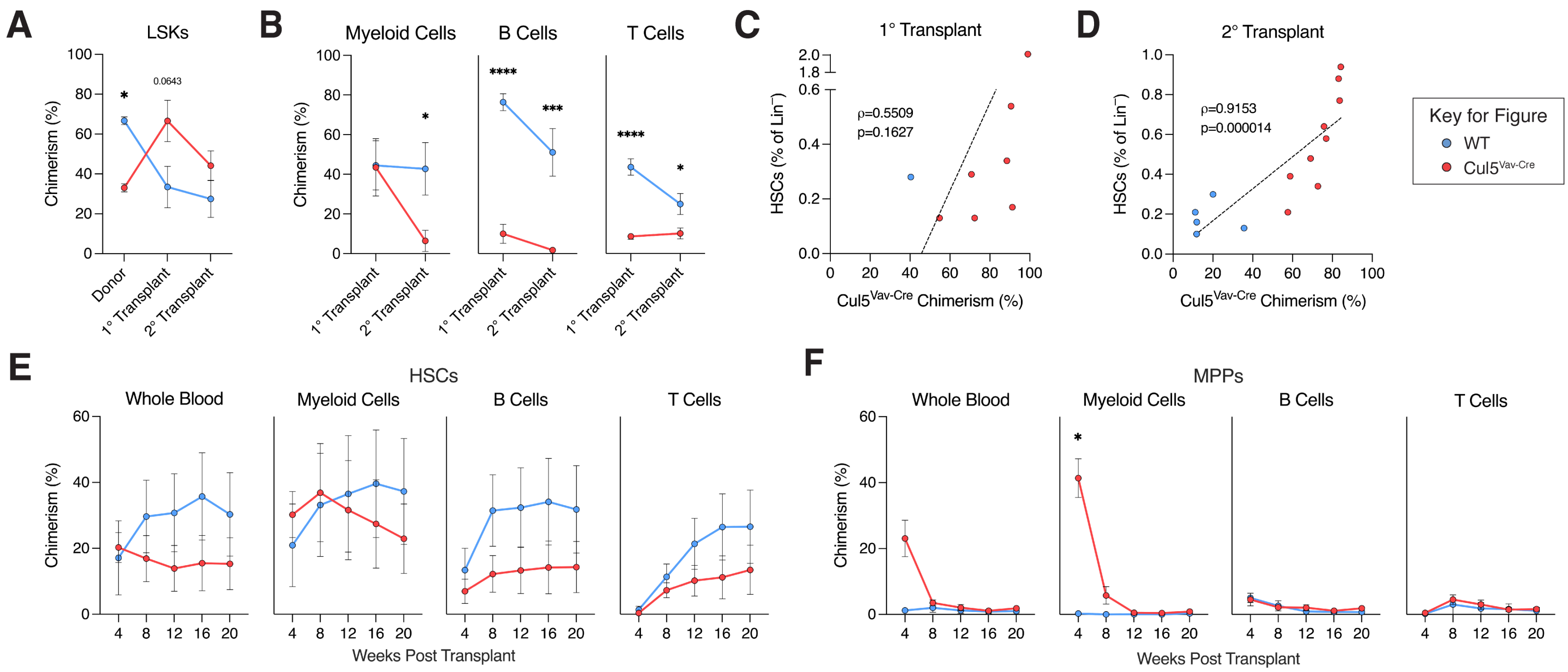

Supplemental Figure 4. Bone Marrow Transplants

Percent chimerism of LSKs (**A**) and mature lineage cells (**B**) in competitive bone marrow transplants at 15 weeks. $Cul5^{Vav\text{-}Cre}$ HSC chimerism by percent of HSCs in the bone marrow in primary (**C**) and secondary recipients (**D**) at 15 weeks. (p=significance value, ρ=Spearman coefficient) (n=3 donor pairs, n=8 primary recipients, n=14 secondary recipients). Percent chimerism in whole blood, myeloid cells, B cells and T cells in sorted HSC (**E**) or MPP (**F**) competitive transplants at 16 weeks (n≥3). Male and female donor mice of the following ages were analyzed: (A-D) 12-13 weeks; (E-F) 12-20 weeks. The following tests were used to determine significance: (A, B, E and F) Unpaired t-test with Holm-Šídák correction; (C and D) Spearman correlation. (ns <0.1 * p<0.05; ** p<0.01; *** p<0.001; **** p<0.0001)

A

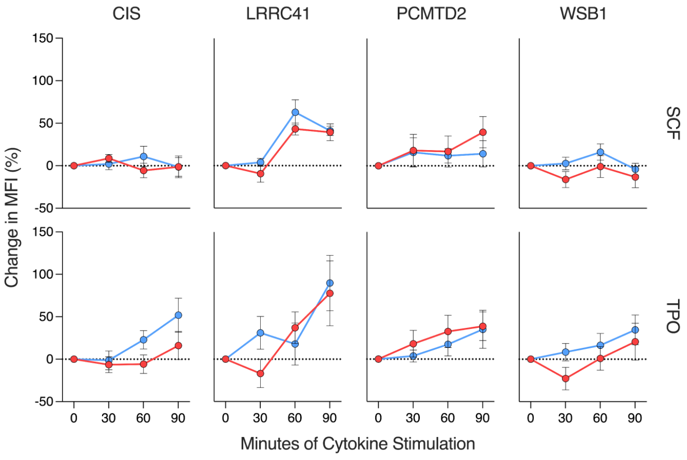

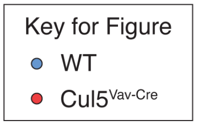

D

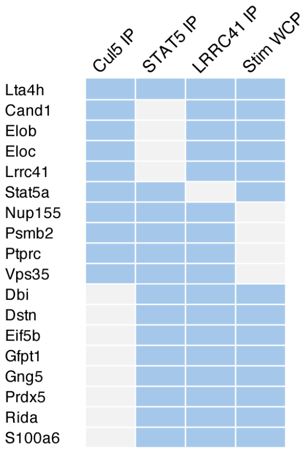

B

LRRC41 IP

Gene Name	Gene Abbr	Combined Peptide Counts: LRRC41 IP	Combined Peptide Counts: IgG IP
Leucine-rich repeat-containing protein 41	Lrrc41	39	0
Elongin-B	Elob	4	0
Elongin-C	Eloc	7	0
Proteasome subunit alpha type-5	Psma5	5	0
Proteasome subunit alpha type-2	Psma2	2	0
Cullin-associated NEDD8-dissociated protein 1	Cand1	1	0
Cullin-5	Cul5	1	0
Ubiquitin-like protein NEDD8	Nedd8	1	0
Proteasome subunit alpha type-4	Psma4	1	0
Proteasome subunit beta type-10	Psmb10	1	0
Proteasome subunit beta type-2	Psmb2	1	0
26S proteasome regulatory subunit 7	Psmc2	1	0
26S proteasome regulatory subunit 8	Psmc5	2	0
26S proteasome regulatory subunit 10B	Psmc6	1	0
26S proteasome non-ATPase regulatory subunit 6	Psmd6	1	0

E

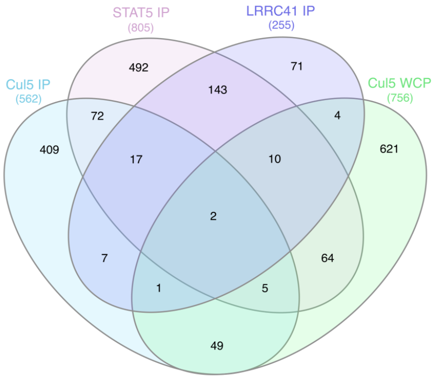

C

STAT5 IP

Gene Name	Gene Abbr	Combined Peptide Counts: STAT5 IP	Combined Peptide Counts: IgG IP
Signal transducer and activator of transcription 5A	Stat5a	368	6
Signal transducer and activator of transcription 5B	Stat5b	121	4
Proteasome subunit alpha type-5	Psma5	2	0
Proteasome subunit alpha type-4	Psma4	1	0
Proteasome subunit beta type-2	Psmb2	1	0

Supplemental Figure 5. LRRC41 and STAT5 IP

(**A**) Percent change in MFI of substrate receptors in WT and Cul5$^{Vav\text{-}Cre}$ HSCs stimulated with SCF or TPO (n≥3). Peptide count of proteins co-immunoprecipitated with LRRC41 (**B**) or STAT5 (**C**). List (**D**) and Venn diagram (**E**) of proteins co-immunoprecipitated with CUL5, STAT5 and/or LRRC41, and proteins increased in Cul5$^{Vav\text{-}Cre}$ LSKs over WT. Male and female mice of the following ages were analyzed: (A) 22-55 weeks; (B-E) 11-19 weeks. The following tests were used to determine significance: (A) Unpaired t-test with Holm-Šídák correction. (ns <0.1 * p<0.05; ** p<0.01; *** p<0.001; **** p<0.0001)

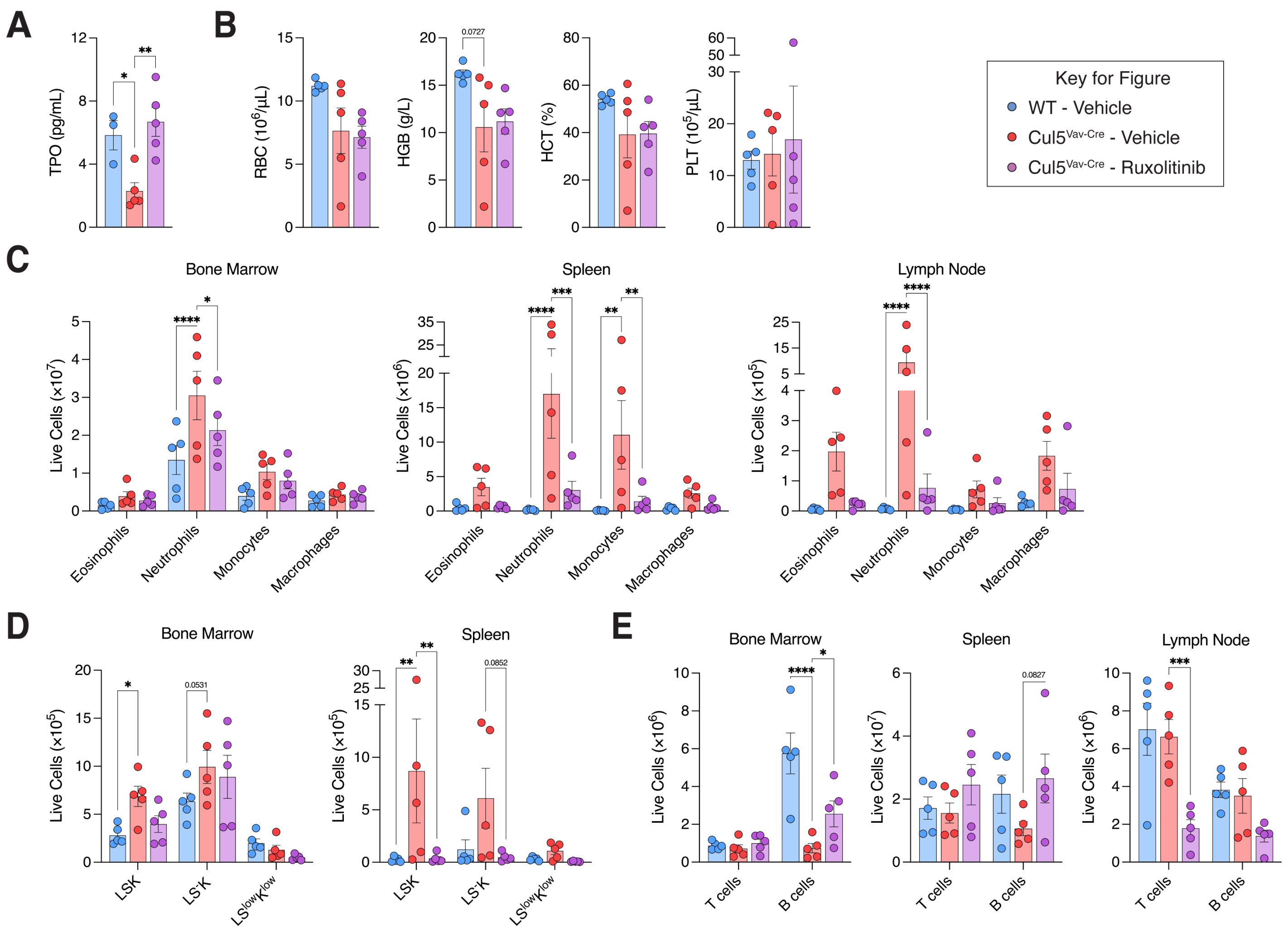

Supplemental Figure 6. Ruxolitinib-Treated Cul5$^{Vav\text{-}Cre}$ Mice The following groups of mice were used in these figures: WT vehicle, Cul5$^{Vav\text{-}Cre}$ vehicle and Cul5$^{Vav\text{-}Cre}$ ruxolitinib treated (n=5/group). (**A**) TPO levels in serum. (**B**) CBC values. Live cell numbers of myeloid populations (**C**), Lin^{-} populations (**D**), and lymphoid populations (**E**) in bone marrow, lymph node and/or spleen. Male and female mice, aged 10-30 weeks analyzed. Two-way ANOVAs with Holm-Šídák correction were used to determine significance. (ns <0.1 * p<0.05; ** p<0.01; *** p<0.001; **** p<0.0001)
